# Sleep Alters the Velocity of Physiological Brain Pulsations in Humans

**DOI:** 10.1002/advs.202503745

**Published:** 2026-02-03

**Authors:** Ahmed Elabasy, Heta Helakari, Tommi Väyrynen, Zalán Rajna, Niko Huotari, Lauri Raitamaa, Ville Isokoski, Matti Järvelä, Mika Kaakinen, Johanna Piispala, Mika Kallio, Vesa Korhonen, Tapio Seppänen, Vesa Kiviniemi

**Affiliations:** ^1^ Oulu Functional Neuroimaging, Research unit of Health Sciences and Technology Faculty of Medicine University of Oulu Oulu Finland; ^2^ Radiology, Department of Diagnostics Medical Research Center Oulu University Hospital Oulu Finland; ^3^ Oulu Center for Cell‐Matrix Research Faculty of Biochemistry and Molecular Medicine Biocenter Oulu University of Oulu Oulu Finland; ^4^ Center for Machine Vision and Signal Analysis University of Oulu Oulu Finland; ^5^ Clinical Neurophysiology, Department of Diagnostics Oulu University Hospital Oulu Finland; ^6^ Clinical Neurophysiology, Research unit of Health Sciences and Technology Faculty of Medicine, Medical Research Center University of Oulu

**Keywords:** CSF oscillatory flow, glymphatic system, MREG, physiological brain pulsations, sleep, ultrafast fMRI

## Abstract

**Introduction**: The clearance of brain metabolites increases during sleep, in association with increased spectral power of the three main cerebrospinal fluid (CSF) flow drivers: cardiovascular, respiratory, and vasomotor brain pulsations. However, little is known about how the increased power of these pulsations affects the velocity and direction of fluid flow in the sleeping brain.

**Objectives**: To address this knowledge gap, we mapped the CSF oscillatory flow velocity in relation to the changing physiological pulsations in the brains of 22 healthy volunteers during sleep and waking.

**Methods**: We used the ultrafast magnetic resonance imaging sequence known as magnetic resonance encephalography (MREG) for tracing the pulsatile movement of water molecules inside the cranium. First, we conducted a phantom validation study with optical flow analysis to confirm that MREG accurately tracks pulsatile water molecule flow in a porous tissue medium. Next, we obtained MREG recordings for mapping the three physiological pulsations without aliasing in the human brain across the awake and sleep states; we thereby quantified the brain‐wide 3D velocity $\underset{V}{\to }$ vectors (i.e., the velocity v_s_ and 3D direction $\hat{v}$) of each pulsation band, using comprehensive dense optical flow analysis during EEG‐verified sleep in comparison to the awake state. Finally, we assessed relationships among the spectral power of the physiological pulsations, their 3D velocity $\underset{V}{\to }$, and slow‐delta EEG power, which is known to depict the increased interstitial volume during sleep.

**Results**: In our phantom study, dense optical flow analysis reliably detected water flow in tissue driven by external pulsations. In healthy volunteers, sleep increased flow velocities ($\underset{V}{\to }$) of the pulsations by more than 20% in concert with elevations in respiratory pulsations and vasomotor waves, while the velocity of cardiovascular pulsations (v_s_) declined by the same percentage. There was a significant anticorrelation between cardiac mean spectral power and slow delta EEG mean power, and a significant correlation between vasomotor mean spectral power and slow delta EEG mean power over the whole brain.

**Conclusions**: Phantom studies validated the optic flow analysis of fast MREG recordings. Sleep altered the 3D velocity dynamics of all neurofluidic brain pulsations in a manner consistent with increased interstitial space and greater fluid exchange, thus supporting the glymphatic model wherein physiological pulsations drive bulk flow during sleep.

## Graphical abstract

Sleep alters I/CSF oscillatory flow, driven by increased respiratory (29%) and vasomotor pulsation (21%) velocities, while cardiovascular pulsations decreased by (22%). Velocity is quantified using optical flow analysis of MREG data. Spectral power increases alongside these pulsations (spatial correlation, r = 0.35 and r = 0.39, respectively), revealing dynamic CSF flow analogous to the correlation between flow velocity and power in a porous medium.

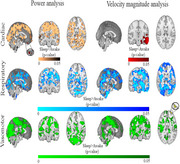

## 1 Introduction

The human brain consists of about 80% water, which can exchange dynamically between the vascular, cerebrospinal fluid (CSF), interstitial fluid (ISF), and intracellular fluid compartments [1]. The glymphatic system is a perivascular pathway responsible for water exchange between these compartments and convective flow in the I/CSF compartments, which is driven by the physiological brain pulsations [2, 3] and osmotic gradients arising from neuronal activity [4, 5]. In anatomic terms, the glymphatic pathway enables the flow of CSF admixed with the interstitial ISF along perivascular spaces embedded between the basement membrane and glia limitans of the BBB [6]. The CSF flow through the brain parenchyma increases during sleep, carrying with it soluble metabolic waste products that have accumulated in the ISF during wakefulness, and ultimately finding egress from the brain through conventional meningeal lymphatics and perinervous connections [6]. While CSF, ISF, and intracellular compartments can readily interchange water, the blood water remains relatively isolated by the blood brain barrier (BBB). However, since water inside the cranial vault is incompressible, any pressure and/or volume changes in one of these brain fluid compartments will induce an opposite change in the other compartments, in accordance with the Monro‐Kellie doctrine. Thus, any pulsatile activity in the vascular or CSF compartments will transfer to movement of water molecules inside the other intracranial compartments, (Figure S10) [7, 8, 9]. There is scant documentation of these phenomena in relation to the transition between awake and sleep states.

Researchers have primarily visualized I/CSF fluid movement using two invasive imaging techniques. The first approach entails injecting fluorescent tracers into the CSF spaces and monitoring their movement with multi‐photon microscopy, a high‐resolution method suitable for imaging within the living rodent brain [3, 10]. The second approach entails dynamic magnetic resonance imaging (MRI) after intrathecal injection of gadolinium‐based contrast agents to map the macroscopic flow of I/CSF, predominantly in the cerebral cortex in rodents [6, 11] and humans [12, 13, 14], which reveals the spatial patterns of bulk fluid flow throughout the brain on time‐scales extending from minutes to hours. However, due to scaling effects in the large human brain, contrast‐based MRI methods primarily capture slow net fluid movement, and are thus unfit to depict the three main physiological drivers of I/CSF flow, i.e., cardiovascular and respiratory pulsations, along with very low‐frequency (VLF < 0.1 Hz) vasomotor waves.

Recent findings suggest that each of these three pulsation mechanisms contributes separately to propelling I/CSF flow through the glymphatic pathway [5, 11, 15, 16, 17, 18]. Studies in living mice showed that cardiovascular pulsations drive the movement of solutes along the peri‐arterial CSF spaces embedded in the cortical subarachnoid space [6, 11, 13]. In contrast, respiratory pulsations primarily drive downstream CSF flow in the (peri)venous spaces [7, 19, 20, 21]. The VLF vasomotor waves from arteries and arterioles have recently been proposed to contribute to solute transport by promoting I/CSF convection in addition to effects mediated by the regulation of local perfusion rate [7, 22, 23]. Previous rodent work has also detected a shift in electroencephalogram (EEG) spectral power from faster rhythms to the slower delta band during sleep in causal association with increased brain solute transport [24], lending further support to the critical importance of the solute transport in altering brain interstitial electrolyte concentrations that govern neuronal activity. During human sleep, the spectral powers of all three physiological brain pulsations increase in concert with increased slow delta EEG activity in overlapping brain areas as a function of sleep depth [25, 26].

In contrast to tracer methods, ultrafast functional MRI (fMRI) utilizing magnetic resonance encephalography (MREG) sequences enables the non‐invasive tracking of water molecules within the several intracranial compartments noted above by following magnetic spin perturbations in water protons. This technique can simultaneously quantify and disentangle the macroscopic signals corresponding to the three physiological pulsations that propel blood flow and I/CSF flow [7, 9, 27]. Importantly, the MREG technique critically samples the physiological pulsations at an ultrafast 10 Hz (i.e., 10 whole 3D brain scans per second) sampling frequency of the T2* weighted brain signal. This enables the accurate acquisition and separation of ca. 1 Hz (peri)arterial cardiovascular pulsations, 0.3 Hz respiratory pulsations, and < 0.1 Hz VLF vasomotor waves of brain arteries, all without aliased mixing of the three pulsations. This presents a distinct advantage over classical fMRI, which irretrievably aliases the physiological pulsations over the low frequency range of the BOLD signal due to the non‐critically low image repetition time (TR), which exceeds 500 ms [7, 28, 29].

Optical flow analysis is a method for quantifying inherent movement, which was originally developed to measure motion between successive 2D planar images [30]. Aiming to quantify movement in brain pulsations, we extended the framework of optical flow analysis for 3D depiction of pulsatile motion within the human brain MREG data [31]. This approach allowed quantification of the velocity dynamics in both CSF and blood flow driven by cardiovascular brain pulsations originating from arterial territories in Alzheimer's disease patients [32], and patients with epilepsy [33]. In a recent application of the optical flow approach, Nenert et al. [34] demonstrated significant associations between physiological brain pulsations, sleep architecture, cognitive performance, and memory consolidation in cognitively normal older adults, suggesting its applicability for mapping brainwide fluid flow across the sleep‐wake cycle.

In general, optical flow can be implemented in two principal approaches: sparse and dense. Sparse optical flow tracks a limited key feature within the scene (e.g., the license plate in a road video of a moving car), whereas dense optical flow computes motion vectors for all pixels (e.g., the whole car in motion), thereby providing a more comprehensive characterization of motion across the entire video image [35]. In our earlier studies, we used a sparse approach for tracking the nadir or peak of physiological signals [32, 33], which might have missed features relative to the dense optical flow approach.

In this study, we first verified in a water flow experiment ‐ using a pineapple fruit as the phantom ‐ that MREG accurately captures water flow in a biological tissue in response to an externally‐applied pulsation. We then extended the phantom study to compare the accuracies of sparse and dense optical flow methods in quantifying the magnitude of water flow velocity. Given the known associations between sleep stage and physiological pulsation spectral power, we proceeded in the main part of the study to investigate the effect of sleep on the velocities ($\underset{V}{\to }$) of the physiological pulsations in the brain of healthy volunteers. Here, we used dense optical flow analysis in conjunction with ultrafast MREG imaging to quantify and map, for the first time, the differing both velocity magnitudes (v_s_) and macroscopic mean direction of water movement as velocity directions ($\hat{v}$) in both CSF and brain tissue for each of the three physiological brain pulsations in individuals in EEG‐verified awake vs. NREM sleep states. We thereby tested the hypothesis that sleep state would manifest in specific brain‐wide changes in velocity magnitude and direction of water movement in relation to brain pulsations during natural human sleep, as predicted by the glymphatic model of increased fluid and metabolic waste clearance during sleep.

## 2 Results

### 2.1 Flow Velocity Verification Result

Because of the diversity of optical flow endpoints, we first present a brief definition of the various terms and then go on to define each parameter: $\underset{V}{\to }$ equals to a vector indicating both the velocity magnitude as length of the vector and direction in 3D (x, y & z) Cartesian space directions of pulse velocity together; v_s_ equals solely to the magnitude of the pulse velocity; $\hat{v}$ refers to the direction of pulse velocity direction in Cartesian coordinates.

**FIGURE 1:**
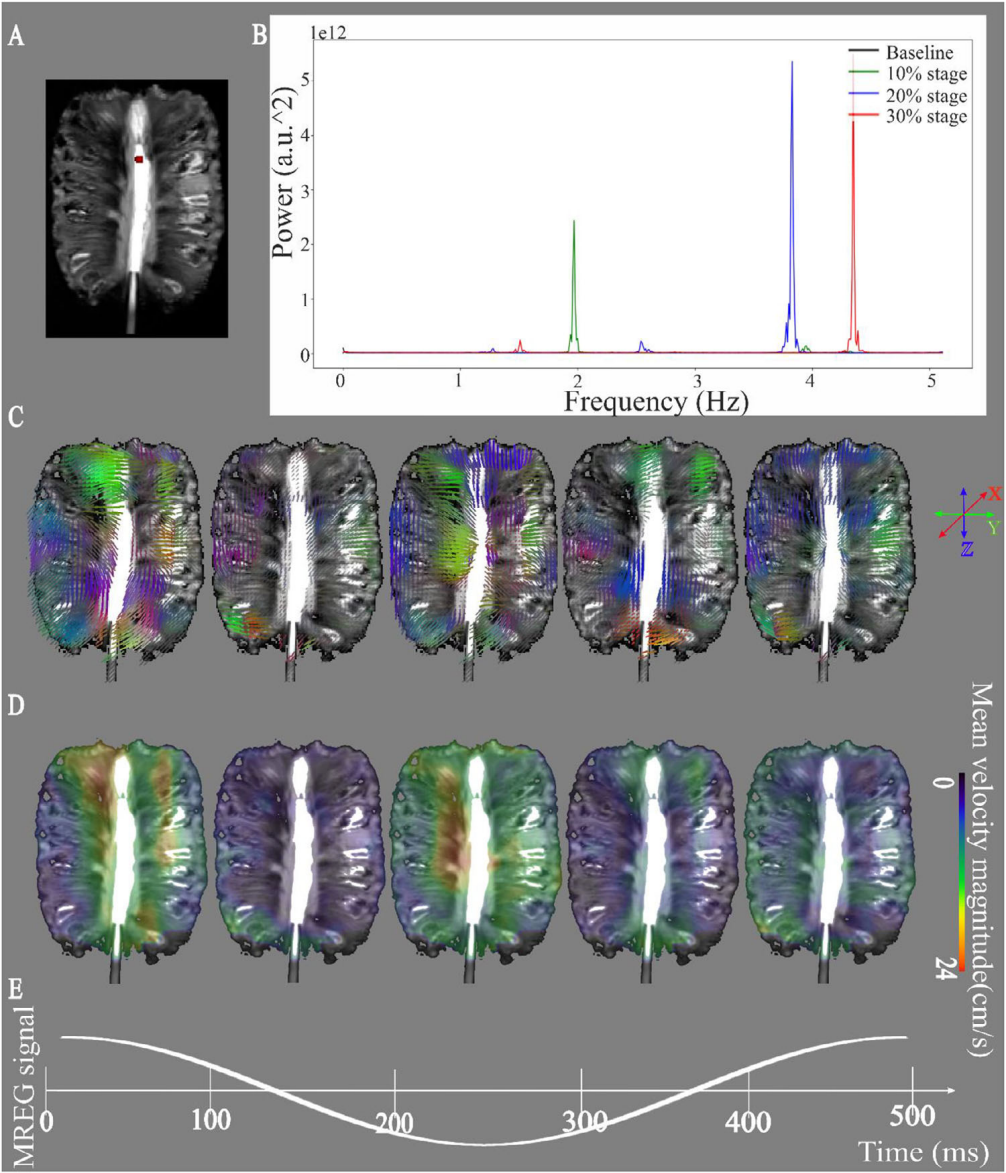
Mean velocity of optical flow analysis at stage 1 pump rotation speed through the pineapple fruit phantom. (A) T2* weighted image of the pineapple. The red voxels indicate the site for measuring the v_s_ of water flow directly in front of the point of entry at the apex of the fruit. (B) An FFT power spectrum of the MREG data accurately depicts the water pump frequencies that were used for subsequent band‐pass image filtering at the various rates of flow velocity inside the pineapple. (C) The mean 3D directional velocity ($\underset{V}{\to }$) maps for the stage 1 water flow pulse rate in five time‐segments over the 0.5 s (2 Hz) pulsation cycle. The directional vectors tend to align in certain phases with the pineapple flesh structural streaks (D) Mean pulsation velocity (v_s_) analysis of stage 1, over the five time‐segments of the cycle. (E) Representative time domain mean signal of stage 1 measured from the cross‐sectional voxels illustrated in Figure 1A.

To verify the fitness of ultrafast MREG imaging for quantifying pulsatile water flow in a porous medium, we obtained velocity ($\underset{V}{\to }$) measurements indicating both magnitude and direction of water molecule movement within a phantom consisting of a fresh pineapple connected via a bore‐hole to a peristaltic pump delivering water, as illustrated in Figure 1A and Figure S1. This approach is widely used in fMRI and DTI studies, based on the rich “interstitial” radial water movement characteristics through pineapple flesh [27, 36, 37, 38]. We set the Watson‐Marlow 313S peristaltic three‐head pump at different flow speeds, corresponding to 10% (∼2 Hz), 20% (∼4 Hz), and 30% (∼6 Hz) of its maximum speed (i.e., 20.1 Hz). At each of these settings, we observed a distinct peak in the MREG data power spectrum, as shown in Figure 1B and Figure S2. For each incremental pump stage, the spectral power and frequency of the MREG pulsation spectrum also increased, accurately mirroring the increased pulsation spectral power inside the phantom. However, the power spectra indicated typical aliasing of fast pump pulsations from around 6 Hz back to 4.3 Hz, as expected when exceeding the Nyqvist cut‐off value of 5 Hz at the 10 Hz MREG sampling rate, as seen in Figure 1B and Figure S2 [27]. Therefore, we did not further analyze the results from the aliased third speed stage.

There was a direct relationship between power and velocity magnitude (v_s_) inside the porous medium of the pineapple, c.f. Figure 1B. The 3D dense optical flow analysis of v_s_ at 2 Hz can be seen in Figure 1C and at 4 Hz in Figure S3. Corresponding results of sparse optical flow analysis of the same pump speeds are presented in Figures S4 and S5. We emphasize that the velocity directional vectors ($\hat{v}$) detected by dense optical flow analysis aligned with the orientation of the radial pineapple flesh streaks from the midline drillhole toward the hard, impermeable rind (Figure 1C). The flow v_s_ maxima naturally aligned along the borehole direction (Figure 1D). Interestingly, the mean v_s_ across the defined regions of interests (ROIs) exhibited a double‐peak, with maxima during the peak and nadir of the single 2 Hz pulse cycle (Figure 1D).

Table 1 presents a comparison of the maximum velocity peaks in the Doppler ultrasound measurements at the point of entry in the pineapple (c.f. Figure S1) as a ground truth, along with sparse and dense optical flow analyses. To obtain a robust measure of optical flow for the 1‐min data MREG époques, we calculated the mean of all the signal maxima across a cross‐sectional area of the pineapple phantom, located just with the flow entry point (the red voxels in Figure 1A). The results indicated that the dense optical flow shows a more accurate measure of the maximum velocity relative to the Doppler ground truth.

**TABLE 1:** Comparison between analysis methods for measuring optical flow at different maximum velocities as quantified by Doppler ultrasound in the 6 mm inner diameter plastic inflow tube just outside the point of entry into the pineapple, vs. the corresponding flow velocity just inside the entry bore hole marked by the red dot in Figure 1A. The dense optical flow method, which detects all movement changes, had higher accuracy for measuring maximal flow velocity than did the sparse method, which searches only for the peak movement.

Measurement	10% stage (cm/s)	20% stage (cm/s)
Ultrasound	22	33
Dense optical flow	23.6	34.8
Sparse optical flow	13.1	37.8

### 2.2 Population and Sleep Staging Results

We scanned a group of 22 healthy control subjects (27.2 ± 4.9 years, 12 females) during an awake state in the afternoon (4–6 PM) after a normal night's sleep. Twelve subjects (26.2 ± 4.3 years, 5 females) were scanned while asleep (6–8 AM) after a full night of sleep deprivation, and ten further subjects (28.4 ± 5.7 years, 7 females) while asleep during normal sleep time (10–12 PM). We recorded sleep scoring in all participants, and used the 5‐min data epoch with the highest amount of sleep for the flow analysis. We present a study enrollment flow chart in Figure S9.

To ensure the selection of data segments with the highest levels of sleep and wakefulness, two experienced clinical neurophysiologists conducted EEG‐based sleep classification in accordance with the American Academy of Sleep Medicine (AASM) guidelines for awake state and the N1, N2, and N3 stages on non‐rapid eye movement (NREM) sleep. This evaluation provided percentage distributions of scan time across different arousal states during the collective sleep recordings, enabling precise identification of the most representative epochs for further analysis: Awake 10%, N1 stage 43%, N2 stage 43%, N3 stage 1%, artifacts 2%. Therefore, 87% of the selected sleep scan époques were scored as NREM sleep. Corresponding values for awake scans were: Awake 99%, N1 stage 0.5%, and artifacts 0.5%.

### 2.3 Human Brain Cardiovascular Pulse Velocity

We bandpass‐filtered the MREG data to select the cardiac frequency band, setting the lower cutoff at 0.51 Hz to capture the minimum cardiac pulsation frequency of all subjects, based on the global minimum of Cardiac SpO_2_ signal of all subjects. To preserve all relevant cardiac harmonics, the upper cutoff was set at 5 Hz, a choice informed by our previous findings, which demonstrated that the cardiac signal together with its harmonics most accurately follow the cardiac pulse signal [32, 39], i.e., exhibiting a distinct fast systolic dip followed by slow diastolic rise after the arrival of the arterial pulse in the anterior cerebral artery (ACA) [32, 39]. Following the filtering step, we applied dense optical flow analysis to the data, consistent with the methodology used in our phantom experiment. We resampled all cardiac pulsations to have the same duration of 0.9 s.

**FIGURE 2:**
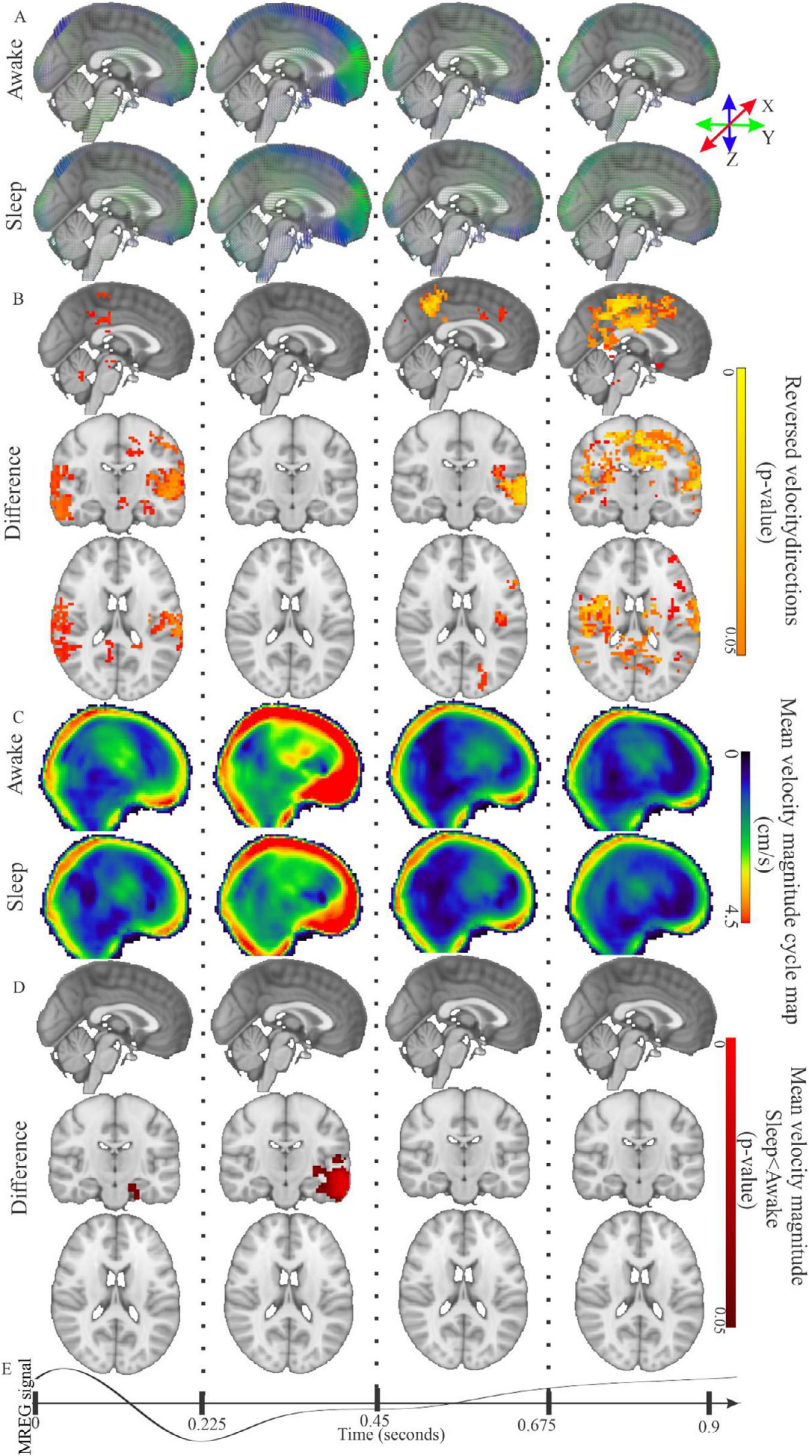
Optical flow analysis of group‐mean cardiovascular brain pulsations in awake and sleeping subjects detects slower velocity and reversed directionality. (A) Awake and NREM sleep group mean (*n* = 22) 3D mean $\underset{V_{\mathrm{card}}}{\to }$ midline sagittal maps with combined velocity magnitude (v_s_) depicted as vector length and direction in color in the MNI 3D x, y & z directions of the cardiovascular pulse in four separate time segments over the group‐averaged 0.9 s cardiac cycle. (B) Three‐plane maps of statistically significant (randomise FWER correction, *p* < 0.05) reversal in the directional vectors $\hat{v}$_card_ in NREM sleep (*n* = 22) during the systolic and late diastolic part of the cardiovascular cycle (hot yellow) in NREM sleep. (C) Awake and NREM sleep group‐mean (*n* = 22) sagittal midline velocity magnitude (v_s_card_, cm/s) maps. (D) Statistical analysis (randomise FWER correction, *p* < 0.05) maps indicate significant v_s_card_ velocity reduction of the cardiovascular pulse in early diastole in temporomedial areas. (E) A representative time‐domain cardiovascular signal from the anterior cerebral artery (ACA) from the group‐averaged MREG data (*n* = 22). For a more dynamic view, see also the 3‐plane dynamic video of cardiac pulse velocity in Video S1 and Figure S6 for summed total volume of changes over the cardiac cycle.

The averaged group level 0.9 s dense optical flow results of the human cardiovascular brain pulse cycle showed a highly dynamic maximal pulse velocity $\underset{V_{card}}{\to }$ at the arrival of every heartbeat to the ACA (Figure 2A,C), occurring approximately 0.3 s after the cardiac R peak on the electrocardiogram (ECG), which is identical to previous findings in healthy control and Alzheimer's disease patient populations [32].

Overall, the cardiovascular pulse velocity magnitude (v_s_card_) in the whole brain was the fastest of the three physiological brain pulsations, with a group average whole brain v_s_card_ (*n* = 22) of 3.27 ± 0.63 cm/s and a maximum of 9.39 cm/s for the awake state. For the NREM sleep state, the group average v_s_card_ (*n* = 22) over the whole brain was 2.98 ± 0.67 cm/s, and the maximum v_s_card_ 7.41 cm/s. The mean v_s_card_ was thus 22% higher in the awake state compared to the sleep state in the regions showing significant changes in Figure 2D. The spatial 3D velocity of the cardiovascular pulsation ($\underset{V_{card}}{\to }$), along with changes in the velocity direction of cardiac pulse ($\hat{v}$_card_), are presented in Figure 2A,B. For more comprehensive, 3D dynamic views of the velocity changes, see Video S1.

Statistical comparison of the direction of propagating cardiovascular brain pulse vectors showed significant directionality alterations in $\hat{v}$_card_ during the early systolic and late diastolic quarters, with reversal of the pulse directionalities, c.f. Figure 1A,B. Also, the scalar v_s_card_ was significantly lower in the left temporomedial brain areas during the systolic part of the cardiovascular pulse in NREM sleep vs. awake state (Figure 1C,D).

Overall, the group mean v_s_card_ (*n* = 22) were somewhat higher than 3 cm/s in grey and white matter and in the pre‐pontine CSF around the basilar artery, as compared to the 2 cm/s for CSF spaces in the ventricles and around the cerebral aqueduct (Figure S7A). The mean directional STD of ${\hat{v}}_{\mathrm{card}}$ over the whole flow cycle for all ROI compartments was close to 1.2 radians, albeit being somewhat lower in CSF spaces, but with higher STD between subjects (Figure S7B).

### 2.4 Respiratory Brain Pulsation Velocity

To select the respiratory frequency band, we bandpass‐filtered the MREG data by setting the cutoffs to the 0.08‐0.49 Hz range, based on the minimum and maximum respiratory rates measured from individual respiratory belt measurements over all subjects. We next applied dense optical flow analysis to the data, following the methodology used in our phantom experiment, and resampled all measured respiratory pulsations to equal segments of 6 s duration.

The whole brain group mean (*n* = 22) respiratory velocity magnitude v_s_resp_ in the awake state was 0.26 ± 0.07 cm/s, with a maximum of 1.10 cm/s, vs. a group mean (*n* = 22) in the sleep state of 0.31 ± 0.01 cm/s (*n* = 22) and a maximum of 1.11 cm/s. The pattern of 3D respiratory pulse in brain was spatially more complex than for cardiac pulse (c.f. Figures 2A), which is best appreciated from comparative viewing the 3D dynamic Videos S1 and S2. The respiratory velocity vectors $\underset{V_{resp}}{\to }$ illustrate the more complex directional of the respiratory pulsation directions in brain tissue.

**FIGURE 3:**
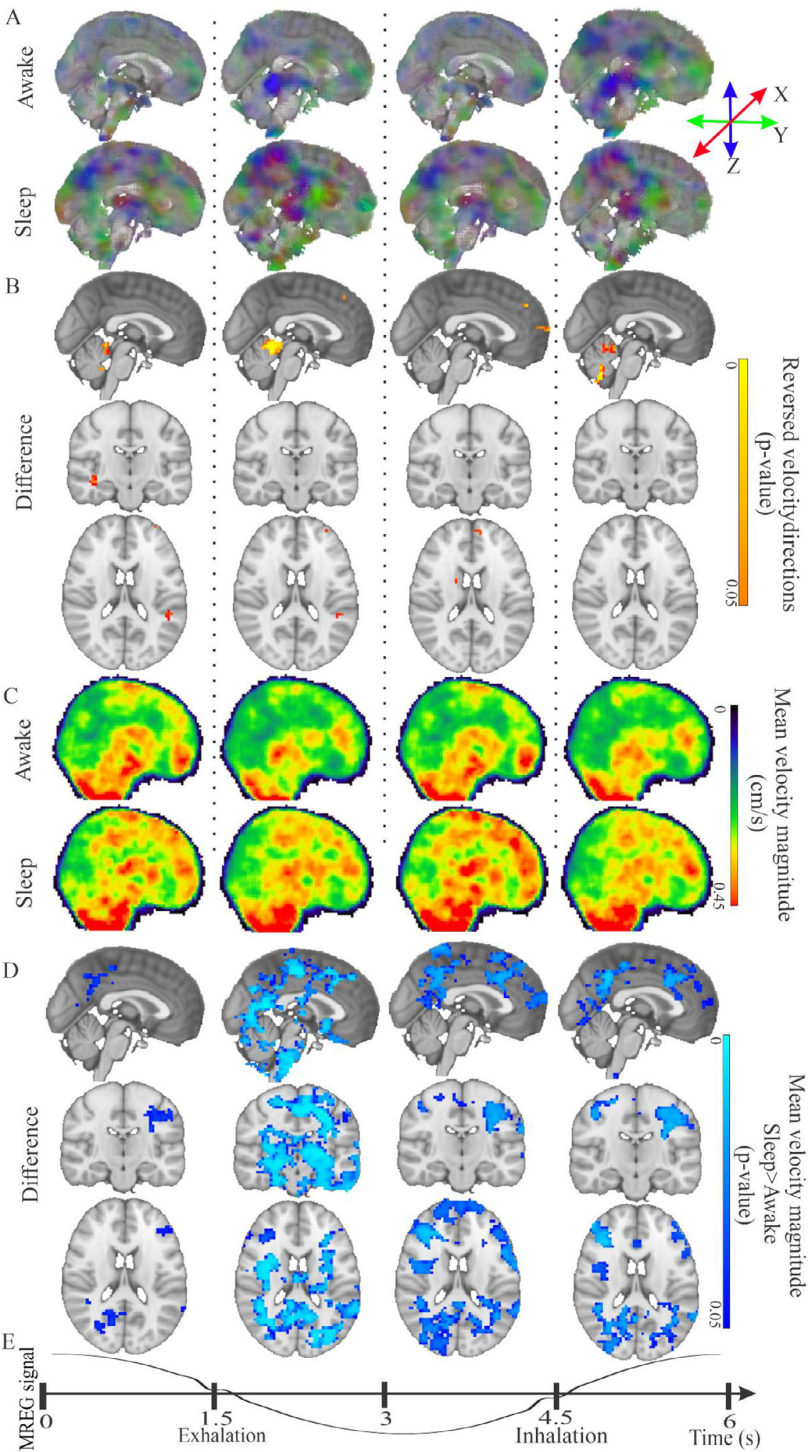
Group mean respiratory velocity increase dynamics in brain of awake (*n* = 22) and NREM sleep (*n* = 22) states. (A) Awake and NREM sleep Mean $\underset{V_{\mathrm{resp}}}{\to }$ across the averaged respiratory cycle (B) Significantly reversed ${\hat{v}}_{\mathrm{resp}}$ to paired statistical comparison between awake and sleep groups (randomise FWER correction, *p* < 0.05) showing reversals mostly confined to the cerebellum. (C) Awake and NREM sleep group mean v_s_resp_ over the complete respiratory cycle. (D) Statistical v_s_resp_ differences (randomise FWER correction, *p* < 0.05) indicate significantly increased velocity in periventricular white matter, cerebellum, brain stem and (sub)cortical especially in latter exhalation phase of the respiratory cycle (E) shown below as measured from the MREG signal (A group mean *n* = 22) from the 4th ventricle resampled to 6 s lengths for both groups. For dynamic velocity changes in 3D, see also Video S2.

The v_s_resp_ was as high as 0.8 cm/s in the basal cranial CSF areas around cerebellum, brain stem, and close to hippocampus. Much like our previous findings in healthy volunteers [33], the averaged v_s_resp_ maximized in a bi‐phasic manner over the respiratory cycle, as also seen in concert with pump pulsations in the phantom experiment (c.f. Figure 1D). The v_s_resp_ reached its maximum during the initial quarter of exhalation and the initial quarter of inhalation, as determined by the mean MREG signal from the 4th ventricle ROI (see also Video S2). The directionality changes were markedly smaller than those of the cardiovascular pulses, mostly occurring in the upper cerebellar areas, c.f. Figure 2B vs. Figure 3B.

In comparison to the awake state, the v_s_resp_ increased significantly (family wise error rate (FWER) correction, *p* < 0.05) in the NREM sleep condition. This increase was more pronounced during the latter half of exhalation and in early inhalation during NREM sleep in comparison to awake state, with only focal increases during the first half of exhalation (Figure 3D). In the NREM state, v_s_resp_ increased close to the sagittal sinus, in frontoparietal cortical areas, and especially in subcortical white matter, dorsal bilateral putamina, thalamus, and hippocampal areas, as well as in the brain stem.

The inspirational v_s_resp_ increases occurring in NREM sleep had a more circumscribed spatial distribution and lower statistical significance compared to the exhalation changes (Figure 3D). During mid/inspiration, the v_s_resp_ increased in subcortical white matter in the superior frontal and parietal cortex, while in the subtentorial basal CSF areas, flow velocity fell back to awake levels (Figures 3D; Figure S6A). There were also small, directional ${\hat{v}}_{\mathrm{resp}}$  changes in the cerebellum and in the CSF spaces below the tentorium, and in small frontotemporal cortical areas (Figure 3B; Figure S6B).

After summing over the whole respiration cycle, the v_s_resp_ increased significantly in distinct areas of the brain (c.f. Figure S6A and Video S2). The mean v_s_resp_ was about 29% higher in the sleep state compared to the awake state in these regions, showing a significant change from Figure 3D. Overall, the summed results over the whole cycle (Figure S6A) showed increases in the brain stem, cerebellum, temporomesial hippocampal areas, thalamus, periventricular white matter ROIs, posterior default mode network (DMN), and primary sensory cortices covering the largest volume of increased pulsation velocity.

Figure S7A depicts segmented mean scalar velocity magnitude v_s_resp_, which tended to be lower in brain tissue ROIs compared to CSF areas. Interestingly, the directional variations (STD) of ${\hat{v}}_{\mathrm{resp}}$ pulsations in the brain tissue fell within only about 1.4 radians, with very little population level variance. On the other hand, the free CSF spaces showed markedly greater directional variation, especially in the 3rd and 4th ventricles and in the cerebral aqueduct (Figure S7B).

### 2.5 Vasomotor Brain Wave Velocity

To select the vasomotor wave frequencies with its assumed harmonics, we bandpass‐filtered the MREG data to the range 0.01–0.08 Hz (thus below the 0.1 Hz Traube–Hering waves), based on our previous studies [25, 26]. Next, we applied dense optical flow analysis to the data, following the methodology used in our phantom experiment. We resampled all vasomotor waves to an identical length of 20 s.

**FIGURE 4:**
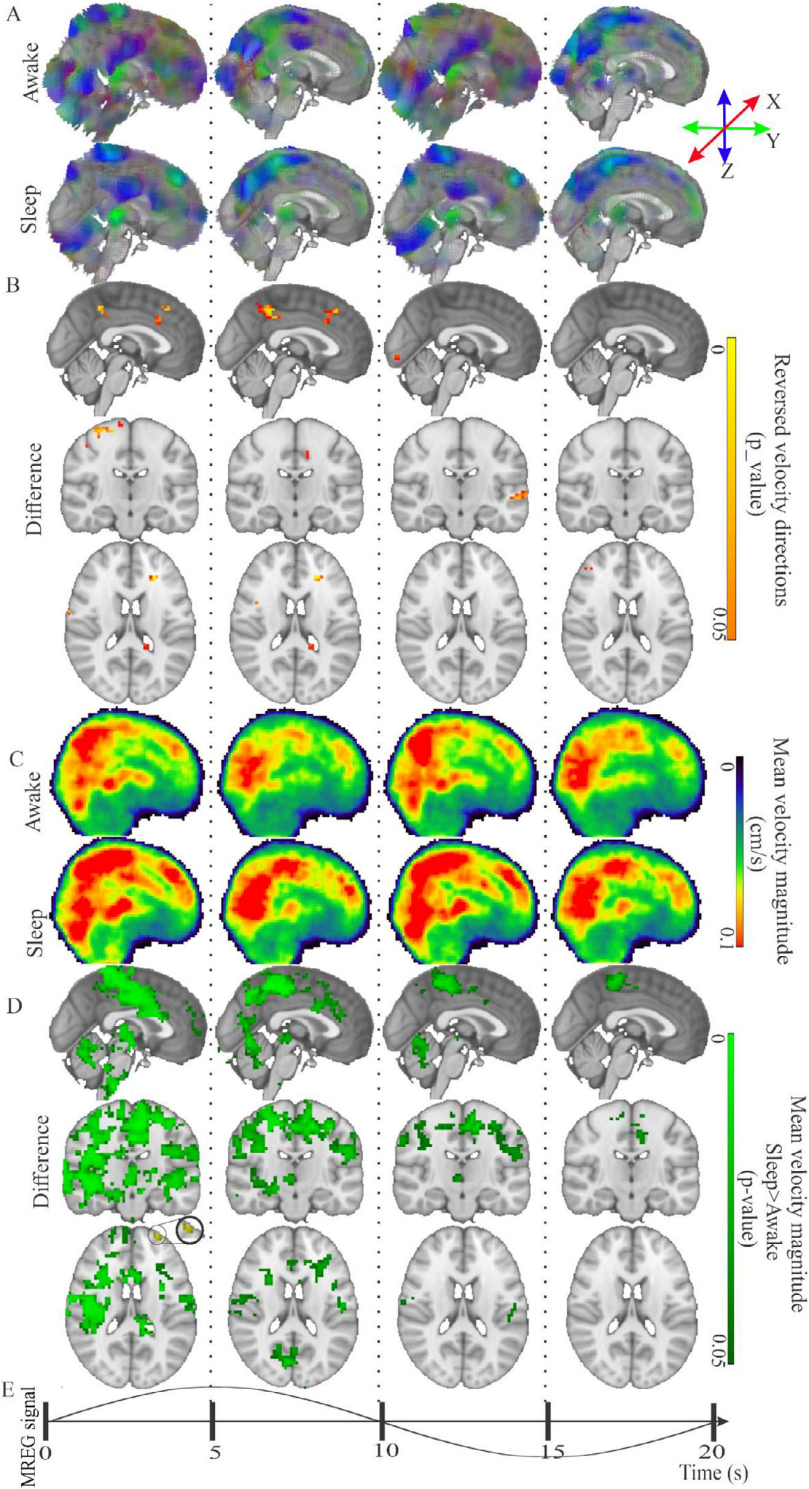
Mean velocity of < 0.1 Hz vasomotor brain waves increases during sleep in specific areas, including sensory cortical and thalamic regions. (A) Awake and NREM sleep group mean $\underset{V_{\mathrm{vaso}}}{\to }$ in awake (*n* = 22) and sleeping (*n* = 22) subjects over a complete vasomotor wave cycle, and (B) reversed velocity direction $\hat{v}$_vaso_ in the awake vs. sleep groups (FSL randomise, FWER corrected, *p* < 0.05), take only midline sagittal again, showing dominantly small midline reversals. (C) Awake and NREM sleep mean velocity magnitude v_s_vaso_ group (*n* = 22) maps, over a complete vasomotor wave, and (D) Three plane statistical difference maps v_s_vaso_ velocity between awake and NREM sleep groups at the four stages of the vasomotor wave (sleep > awake, FWER corrected, *p* < 0.05) indicating a velocity increase at the early BOLD signal increase phase reducing over time. See also Video S3. There is also a small region in yellow (sleep < awake, FWER corrected, *p* < 0.05) indicating an increase in velocity in awake compared to sleep. (E) A group mean representative time domain MREG vasomotor wave signal is shown from the posterior cingulate part of the Default Mode Network. For dynamic velocity changes in 3D, see also Video S3.

The vasomotor waves arising from the posterior cingulate cortex (PCC), which is a component of the default mode network (DMN_PCC_), displayed a distinctly sinusoidal temporal MREG signal pattern (Figure 4E). The vasomotor waves, which are (by definition) low frequency (< 0.08 Hz), had a mean v_s_vaso_ in the awake group of 0.058 ± 0.009 cm/s, and maximal velocity around 0.204 cm/s, i.e., only 2% of the quantifiable maximal cardiovascular band velocity magnitude. In the sleeping subjects, the mean v_s_vaso_ was 0.06 ± 0.01 cm/s, and the maximum 0.253 cm/s across the whole brain. The vasomotor waves presented a rich dynamic 3D spatial pattern, also surpassing in complexity the cardiac pulsations, c.f. Figures 2A and 4A, and Video S3. Like the respiratory velocity magnitudes, v_s_vaso_ also presented a bi‐phasic velocity magnitude maxima pattern during the BOLD signal up and signal down phases of the vasomotor signal. Like the respiratory pulse, the v_s_vaso_ maximized near the mean signal level at the early decrease and increase phases, with steepest MREG signal changes over time (i.e. highest time derivative) in the DMN_PCC_, (Figure 4A,C; Video S3).

The sleep group also showed a significant mean 21% increases in velocity magnitude of the vasomotor wave, occurring predominantly at the initial BOLD signal increase phase of the wave. However, there was some velocity magnitude increase over the entire cycle length in specific brain locations (c.f. Figure 4D), mainly in regions encompassing the primary sensory cortices, frontal cortex, and bilateral thalamus. The hippocampi and especially the cerebellum, lying close to the 4th ventricle showed right‐dominant v_s_ increases in the sleep condition.

Dynamically, the higher v_s_vaso_ in the sleep condition was most distinct during the 1st quarter of the vasomotor wave cycle, extending over the primary sensory cortices. The v_s_vaso_ increase during sleep extended from sensorimotor and associative areas into the midline cingulate gyrus, bilaterally in the pallidum, amygdala, and hippocampus, the midline thalamus, and in right temporal areas. The right cerebellum, brain stem, and 4th ventricle also showed notably higher v_s_vaso_ compared to the awake condition.

Toward the end of the averaged 20 s vasomotor cycle, a transient increase of the v_s_vaso_ tended to normalize in frontobasal and brain stem areas, but an increase over the somatosensory areas and upper cerebellum persisted during some three quarters of the complete vasomotor wave cycle (Figure S6). There was a locus of continuous velocity increase in the juxtapositional lobule in midline parasagittal areas; for dynamics, see the 3D video in Video S3.

In segmented ROI analyses, v_s_vaso_ was higher during sleep in the 3rd ventricle and cerebral aqueduct, albeit with high variance compared to other segmented brain structures (Figure S7E). The CSF spaces showed increased STD of directional variance in the velocity vectors, similarly to respiratory pulsations in comparison to the tight 1.4 radian directionality STD measured in brain structures (Figure S7F).

### 2.6 Power vs. Velocity of the Physiological Brain Pulsations

**FIGURE 5:**
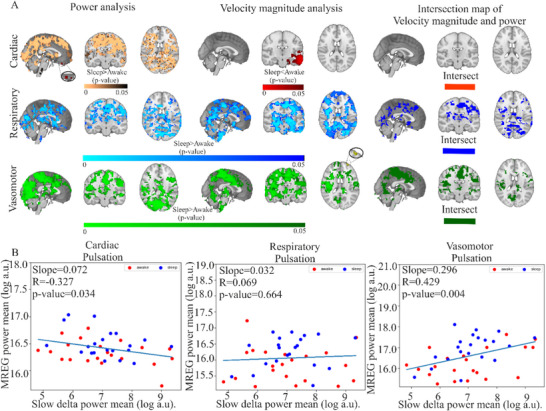
FFT Power vs. optical flow velocity analysis of MREG data and dcEEG slow delta power analysis of (*n* = 22 each) awake and sleeping states. (A) Three‐plane brain maps of increased (FWER, *p* < 0.05) areas of increased FFT power of the pulsations in sleep > awake; top is cardiac (with a small brain region in red showing the converse sleep < awake), middle respiratory and bottom vasomotor (with a small brain region in yellow showing the converse sleep < awake) areas of increased pulsation power. Presented in similar order from top to bottom, the summed spatial maps of decreased cardiovascular pulsation velocity and increased respiratory pulsation and vasomotor wave velocity. Intersection analysis of the power and velocity analyses revealed no overlap in the cardiovascular pulses with increasing power and decreasing velocity, but a spatial overlap in respiratory and vasomotor frequencies with the power and velocity increases. (B) Slow delta EEG power correlated negatively with cardiovascular power, respiratory power had no correlation, and vasomotor power correlated positively with slow delta mean power. Of note, the measured velocity did not correlate significantly with the slow Delta EEG power, c.f. Figure S8.

We have previously detected significant power increases in all three physiological brain pulsations during sleep [25, 26], which we now confirm (Figure 5A). On the other hand, we detected a velocity reduction in sleep compared to awake state in the cardiac pulsations, and an increase in the respiratory and vasomotor pulsation velocities. There was a widespread spatial overlap and covariance of the results of velocity magnitude and power of the slower physiological pulsations in sleep, with Pearson correlation coefficients for the respiratory (r = 0.35) and vasomotor (r = 0.39) pulsations. However, in comparing the two arousal states, the power increases during sleep had a wider spatial distribution compared to the velocity magnitude changes, especially for the low frequency pulsations.

Neurophysiological delta power increased during sleep in mouse brain areas showing increased glymphatic solute transport [3] and in human brain, physiological pulsation power likewise increased during sleep in brain regions showing higher neurophysiological slow delta power [25, 26], with linear correlations between cardiorespiratory power and slow‐delta powers in awake and sleeping states [40]. Therefore, we further investigated whether the concomitant power increases in the physiological pulsations during sleep would likewise mirror slow delta EEG power changes. Our analysis showed that the slow delta EEG power increases in the sleep state correlated significantly with the simultaneous whole‐brain spectral power of vasomotor waves, but did not correlate with respiratory pulsations (c.f. Figure 5B). On the other hand, whole‐brain mean spectral power of cardiac waves anticorrelated with the slow delta EEG power. The pulsation velocity results had no clear association with the slow delta power, neither in the whole brain, nor in the regions showing increased velocity magnitude, c.f., Figure S8.

## 3 Discussion

We investigated the effect of sleep on the velocity of physiological brain pulsations driving I/CSF flow and blood flow, applying ultrafast MREG brain imaging combined with dense optical flow velocity analysis, and simultaneous EEG recordings. We first validated the accuracy of the dense optical flow method against gold standard Doppler ultrasound readings by mapping water flow in a pineapple phantom experiment. We then proceeded to MREG studies in healthy volunteers, obtaining for the first time brain‐wide 3D velocity maps of CSF pulsatile flow during natural sleep in comparison with the awake state in 22 volunteers. Results demonstrated that the velocity of the primary physiological drivers of CSF flow within the human brain —specifically respiratory and vasomotor brain pulsations—were more than 20% greater in specific brain regions during NREM sleep as compared to awake state, while cardiac‐driven pulsation velocity decreased by about 20%. Furthermore, the 3D directionality of the cardiovascular pulsations had significant reversals over broad brain areas in NREM sleep, while the vasomotor and respiratory waves had only small areas of inverted pulse propagation.

### 3.1 NREM Sleep Alters Physiological Brain Pulsation Power and Velocity

#### 3.1.1 Cardiovascular Brain Pulsation

Cardiovascular pulses have been shown to drive the periarterial space CSF flow primarily in the murine sub‐arachnoid space [11]. We detected in healthy volunteers a significant slowing of the cardiovascular pulse velocity in NREM sleep, along with marked reversals in the direction of pulse movement. This concurs with our earlier findings in hippocampal memory‐related areas of Alzheimer's disease patients [32], which we had attributed to blunted I/CSF fluid pulses from narrowed peripheral arteries. The currently observed reversed pulses have a different phase pattern in the cardiac cycle, which must entail a different mechanism in healthy young subjects. In theory, the inversed pulses could reflect reversal in the net movement of neurofluidic tissue water movement in the sleeping brain; while perfusion continues during sleep, there is a reversal of the water molecule movement in perivascular spaces mediating solute efflux during sleep [15].

#### 3.1.2 Respiratory Brain Pulsation

In a phase contrast MRI study, inspiration was the main promoter of CSF flow at the macroscopic scale, with only small effects mediated by cardiac drive [20]. Similarly, at the microscopic level, venous outflow of the brain is predominantly driven by respiratory inhalations, rather than inflowing arterial pulsations [19]. Upon voluntary deeper and forced breathing, there was increased synchronization of venous flow dynamics and CSF flow in the human brain [41], and deep breathing increased CSF flow velocity [42, 43].

It is well‐established that upper airway resistance, along with pulmonary and intrathoracic ventilation, all increase during sleep [44, 45, 46]. Such changes may have the net effect of also promoting CSF pumping through the perivenous spaces in the brain, which might manifest as increased respiratory pulsatility in MREG. Indeed, our previous MREG results also indicated that the respiratory brain pulsation power increased as a function of sleep depth [25, 26]. Similarly, another BOLD study showed that voluntary deep inspirations increased the magnitude of CSF pulsations, and evoked widespread reactive BOLD signal changes in the cerebral cortex [47]. Increasing end expiratory pressure promotes glymphatic solute transport in subcortical regions of anesthetized mouse brain, but not in the cerebral cortex [48]. In the present human study, we likewise saw sleep‐related increases in velocity magnitude and power of respiratory brain pulsations in overlapping in subcortical and periventricular white matter.

The maximal flow velocities occurred at the midpoints of exhalation and inhalation, but sleep‐induced increases in the respiratory velocity were most pronounced at the latter part of exhalation, albeit the changes prevailed through the whole cycle gradually declined until the end of inhalation. The detected increase in respiratory‐driven water molecule velocity within brain tissue (without marked changes in the directionality) could reflect the known inflation of interstitial space during sleep, which is permissive to faster convection toward the venous side. Also, the recently‐detected asymmetric extensions of perivenous spaces directly into the venous lumen might account for the faster respiratory‐driven fluid movement.

#### 3.1.3 Vasomotor Waves

Tracer studies in sleeping animals have shown increased brain solute convection and clearance [3, 24]. Especially during NREM sleep, slow arterial vasomotor waves increase in velocity, which in turn enhances fluid flow and drives perivascular CSF space volume changes [49]. Declining orexin signaling slows the firing rate of brainstem *locus coeruleus* noradrenergic neurons [27], which begin to drive sleep‐related arousals associated with 0.02 Hz vasomotor waves that are linked to norepinephrine level oscillations [3, 15, 24, 50]. Human BOLD studies have also verified widespread vasomotor changes, which are linked to cholinergic neurons of the nucleus basalis of Meynert [50], with additional contributions from sympathetic drive, especially in NREM light sleep [47, 51]. Present results align perfectly with these physiological/neurochemical findings, and further imply that the increase in VLF vasomotor wave power is a driver for the 21% faster CSF flow in brain tissue during sleep (Figure 4; Video S3). This flow increase could be due to enlarged interstitial spaces [15, 52], and/or increased velocity of vasomotor waves along the arteries, arterioles, and perivascular spaces during sleep [49].

**FIGURE 6:**
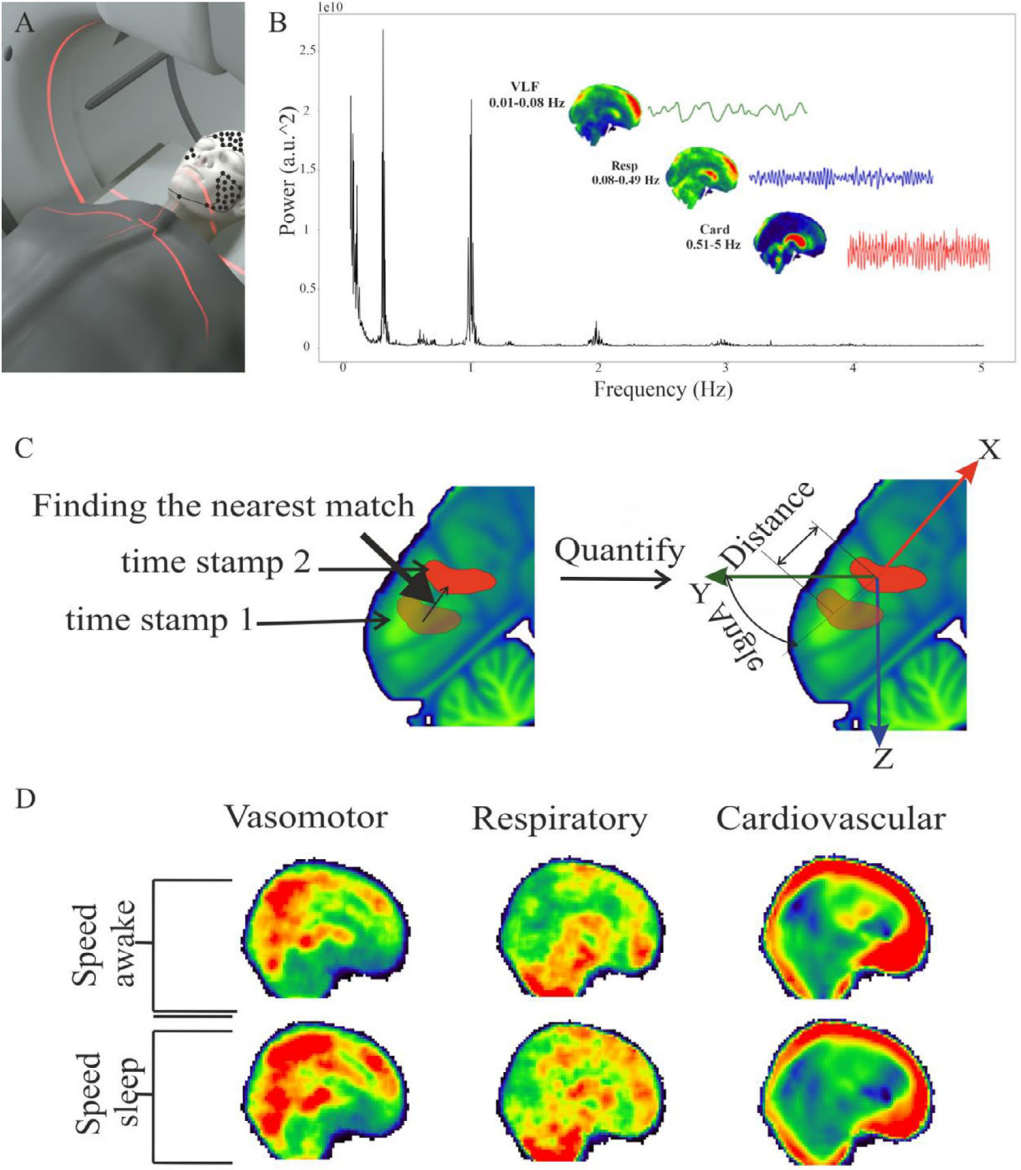
Flow chart for velocity analysis of study subjects. (A) Simultaneous multimodal MREG recordings with a 3T fMRI and dcEEG (EGI ch 256) data collection in awake and sleep states on separate days. (B) FFT power spectral analysis illustrating the distinct signal‐to‐noise characteristics of the vasomotor, respiratory, and cardiovascular brain pulsations. The images illustrate power distributions of these pulsations in MNI152 space. Each pulsation is illustrated on the right after an individual band‐pass filtering of the MREG data to separate the cardiac, respiratory, and vasomotor signals in their own frequency bands. (C) 3D depiction of optical flow analysis of band‐passed data following signal changes between consecutive MREG images obtained 100 ms apart. (D) Visualization of velocity results in MNI space.

Like respiration, the measured velocity of vasomotor waves in the cerebral cortex also maximized during sleep, synchronously with the steepest MREG signal change, as observed in the PCC Default mode network trigger point (Figure 4; Video S3). The vasomotor velocity during sleep maximized during the onset of BOLD signal increase, and was lower during the decreasing phase (Video S3). This indicates that the maximal velocity of the vasomotor pulsation occurred at the vasodilation phase of precapillary arterioles/arteries. Given the recent discovery of perivascular volume pulsations during NREM sleep [49], this vasomotor velocity increase implicates altered perivascular flow and volume in brain tissue. Hydrodynamic resistance of the brain tissue may also reduce during sleep, allowing faster flow through the dilated interstitial spaces. For example, noradrenaline‐mediated widening of astrocytic clefts in the *glia limitans*, and resultant enlargement of extracellular space within the tissue may enable increased flow velocities in sensorimotor cortical areas by decreasing resistance (Figures 4 and 6) [15, 52].

### 3.2 Correlation of Velocity Magnitude with Pulsation Power and Slow Delta EEG Power

We found spatially overlapping patterns of increases in the v_s_resp_ and v_s_vaso_ pulsations during NREM sleep compared to waking. These overlapping regions exhibited increased power and velocity for both pulsations, which correlated with elevated EEG delta power (see Figure 5). Fultz et al. have earlier described the connection between CSF flow fluctuations, BOLD signal changes, and EEG delta power across the human sleep‐wake cycle [4]. That seminal work showed that the CSF flow BOLD signal in the 4th ventricle tracked increasing EEG delta wave activity, while being anti‐correlated with the cortical gray‐matter BOLD signal. Picchioni et al. suggested that, in addition to the triggers arising in the central nervous system, there may be an autonomic pathway for regulating CSF flow in a manner highly dependent on respiration rate [47]. As such, we see a need for better elucidation of the causal relationships between physiological pulsations and arousal state‐dependent electrophysiological signals.

Moreover, we replicated earlier findings of a positive linear correlation of the vasomotor power and slow delta EEG‐power, but found no such significant correlation between pulsation velocities and slow delta EEG‐power, as seen in Figure S8, and as previously reported [40]. Regarding inferred causality, the slow delta wave power may facilitate and accelerate the oscillatory solute flow caused by vasomotor waves. Alternatively, increased delta power could be a *consequence* of enhanced solute transport and osmolality effects, potentially blunting EEG power by expansion of the interstitial space through widening of the inter‐cellular clefts between shrunken astrocytes in the *glia limitans* [15, 53]. Others have suggested that elevated delta power during sleep may result from increased exchange between ISF and CSF in the perivascular spaces [3], with additional contributions from noradrenergic mechanisms as described above [50].

Human sleep experiments have shown that slowly emerging K‐complexes in N2 sleep, and likewise during controlled deep breathing, cause similar CSF inflow changes, which couple to widespread brain BOLD signal changes [47]. In general, wave energy increases with amplitude, enabling faster travel through a porous medium, depending also on the viscous properties of the tissue [54, 55, 56]. The stronger vasomotor waves that occur in NREM sleep [4, 25, 49, 57] could thus mediate increased flushing between I/CSF in the perivascular spaces. We suppose that this phenomenon might dilute local electrolyte concentrations and drop the overall EEG activity toward delta power, in concert with increased water entry into the interstitium.

### 3.3 Velocity Measures in Tissue and CSF Spaces

Transcranial doppler ultrasound (TCD) or phase‐magnetic resonance imaging (pMRI) measurements have indicated flow velocity in the major cerebral arteries typically ranging from 40 to 70 cm/s, and up > 100 cm/s [58, 59]. Such velocities exceed the range of our current optical flow analysis of MREG data, as clearly demonstrated in the phantom experiments (Figure S2). In the human brain cortex and CSF spaces during wakefulness, however, our dense optical flow MREG analysis indicated a mean neurofluidic flow velocity of 3.27 ± 0.63 cm/s in the cardiovascular frequency band, with a maximum flow velocity of 9.39 cm/s in free CSF spaces. These findings closely align with cardiovascular‐driven CSF and venous blood flow velocities (5–8 cm/s) previously measured by Doppler ultrasound, and phase contrast and other MRI techniques in awake humans [60, 61, 62]. Within the constrained space of the cerebral aqueduct, CSF velocities in our 3 mm cubic voxels ranged from 1–5 cm/s, consistent with previous phase‐contrast MRI findings showing mean peak velocities of 3.30–4.08 cm/s during systole [63].

It is technically challenging to measure the substantially lower blood flow velocities in small cerebral vessels using clinical human MRI. Microscopic dynamic imaging of arteriolar erythrocytes in mouse brain tissue indicated velocities up to 22.2 mm/s, with a progressive decrease down to < 1 mm/s at the capillary level, and an increase to 5 mm/s in venules of diameter ranging from 10–60 µm [19]. A single slice high resolution 7T Qflow MRI study found flow velocities ranging from 3.9–5.1 cm/s in the anterior perforating arteries of the human basal ganglia [64]. However, the more abundant literature on transcranial Doppler ultrasound in the mouse ACA gives a reference range of 10–20 cm/s [59, 65], thus one quarter of the present human results. More recent high spatiotemporal resolution 2D ultrasound studies in mice have reported mean blood flow velocities of approximately 5 mm/s (range: 2–7 mm/s) within brain venules [66, 67, 68], thus primarily falling with the range of our velocity for respiratory pulsations (c.f. Figure S7C). Furthermore, human cerebral veins showed transcranial Doppler‐verified mean velocities of 10.1±2.3 (range 4–17) cm/s [69].

CSF velocity in spaces surrounding small arteries of similar size to venules was approximately 18 µm/s in mice [11], which matches the velocity of the present vasomotor waves (Figure S7E). Overall, our observations show a considerable overlap between the CSF and blood flow velocities found by others across different spatial scales and species, suggesting a complex interaction between flows in arterial, venous, and CSF compartments.

It is generally accepted that blood flow to transcranial Doppler sonography declines in cerebral arteries with increasing sleep depth in humans [70]. Indeed, while cardiovascular velocity does decrease, our present findings also revealed significantly *increased* velocities of the slower CSF drivers, namely respiratory and vasomotor waves, during NREM sleep in specific brain regions where the delta power also increased (Figures 3, 4, 5). During sleep, the intracranial pressure increases [71], perhaps due to the increased vasomotor and respiratory spectral power [25].

The pineapple phantom study confirmed that MREG signal data accurately tracks water molecule inflow to a convective vessel and further into a porous tissue medium (Figure 1; Figure S4), which unequivocally responded to increased flow velocities and pump frequency. As a principle of physics, the power (or amplitude) of small‐amplitude or linear symmetric waves has no influence on the velocity in free‐flowing reservoir. However, in a porous medium like the brain, the power of a wave can influence its velocity [54, 55, 56]. More precisely, in the case of non‐linear waves ‐ in the manner of ocean waves arriving into shallowing water at the seashore ‐ the amplitude of the wave does indeed influence its velocity [72]. In the brain, the inflowing blood and CSF are confronted by, respectively, narrowing arteries and peri‐vascular CSF spaces, resulting in non‐linear, asymmetric waves with velocity that is sensitive to effects of pulse amplitude. This model is supported by present findings that vasomotor and respiratory signal changes predominantly occurred within brain tissue, but not in the broader CSF spaces (Figures 3 and 4).

### 3.4 Fluid Movement Imaging with MREG – Strengths and Limitations

In the dense optical flow analysis of the phantom data, increased pulsation spectral power and frequency correlated with increased flow velocities inside the porous pineapple tissue. The dense optical flow method outperformed the sparse method in accuracy, as presented in Table 1.

Our MREG procedure collected T2*‐weighted 3D brain volume images every 100 ms (10 Hz), giving excellent time domain accuracy without hydrodynamic physiological signal aliasing. The fast‐sampling rate imparted greater T1‐weighting of the signal as compared to classical TR 2‐3 sec sampling. As such, the ultrafast MREG signal is also sensitive to fast phase and spin coherence, as well as T1‐weighting signal characteristics. However, the fast sampling sacrifices some spatial detail, such that signals in the 3 mm cubic voxels can arise from multiple sources, i.e., vasculature, CSF, and brain tissue, due to partial volume effects. This means the measured velocities represent a weighted mean of several partially overlapping and often opposing pulsatile flows within each voxel, especially in the cerebral cortex, which has relatively faster arterial inflow and slower venous outflow. Due to this overlap, MREG may underestimate the highest flow velocities due to flow summation, which may be most problematic near CSF/tissue boundaries, where arterial and venous flows are in opposite directions.

Present observations of a double‐peaked velocity pattern within a pulsation cycle (c.f. Figure 1D) calls for some consideration. This pattern is consistent with flow dynamics observed in the present study (Figures 3 and 4), and aligns with previous findings [33, 34]. In the phantom data, such double waves may have been formed from the repeated inflow pulses being followed by a prompt reflective wave from the non‐penetrating pineapple rind along the streaked structural pathway of the fruit's flesh. This may be an analogue for the neurofluidic pulses reflected from in/outflow pathways within the human brain (peri)vascular spaces by the surrounding incompressible cranium.

## 4 Conclusion

This study provides detailed evidence linking increased brain tissue water pulsation velocities. While cardiovascular pulsations velocity declined in sleep, the pulse showed a significant directional reversal inside the brain tissue. In contrast, respiratory and vasomotor pulsations exhibited significantly increased velocities across broad brain regions during NREM sleep. Furthermore, the power and velocity magnitude of the respiratory and vasomotor drivers both increased in areas of increased delta EEG power during sleep, indicating increased water molecule flow in areas of enhanced solute convection. These findings suggest that sleep‐dependent enhancement of brain water flow dynamics that is positioned to play a crucial role in maintaining brain homeostasis, which opens new avenues for developing non‐invasive biomarkers for neurodegenerative and neurological disorders.

## 5 Experimental Section

### 5.1 Subjects

The regional medical research ethics committee of the Wellbeing services county of North Ostrobothnia approved the study (POHDE IRB 118/2012 ‐ 181/2023, FIMEA/2023/005604). All participants gave written informed consent, according to the requirements of the Declaration of Helsinki. We first interviewed and screened prospective participants. Subjects reported good general health, and met the following inclusion criteria: non‐smokers without neurological or cardio‐respiratory disease, no continuous medication, and no possibility for pregnancy. Subjects did not have contraindications or metal in the body that would have precluded MRI scanning. A general pipeline for the velocity analysis of the study subjects in Figure 6.

### 5.2 Data Acquisition

All subjects were scanned in Oulu University Hospital (Finland) using a Siemens MAGNETOM Skyra 3T scanner (Siemens Healthineers AG, Erlangen, Germany) equipped with a 32‐channel head coil. We applied the fast functional magnetic resonance imaging (fMRI) sequence known as MREG [73, 74, 75, 76] in synchrony with a previously described multimodal scanning setup [77]. MREG was a single‐shot, critically sampled (10 Hz sampling rate) sequence that allows visualizing the cardiovascular (≈ 1 Hz), respiratory (≈ 0.3 Hz), and very low frequency (VLF) vasomotor pulsations (< 0.1 Hz), without higher frequencies to alias on the top of the lower frequencies [7].

The following parameters were used for MREG: repetition time (TR) = 100 ms, echo time (TE) = 36 ms, and field of view (FOV) = 192 mm, 3 mm cubic voxels, and flip angle (FA) = 5°. Furthermore, the magnetization crusher gradient between scans was set to 0.1, to avoid masking of the signal by stimulated slow echo drifts, while retaining good sensitivity to physiological pulsations. Parameters for 3D structural T1 MPRAGE were: TR = 1900 ms, TE = 2.49 ms, FA = 9°, FOV = 240 mm, and slice thickness 0.9 mm. We obtained a structural T1 MRI scan in all sessions, for registering the fMRI data to the standard MNI152 3 mm brain template for group analyses.

The Electrical Geodesics (EGI, Magstim Company Ltd, Whitland, UK) MR‐compatible GES 400 system, equipped with a 256‐channel high‐density net, was used to record EEG in synchrony with MREG at settings as previously described [77]. Respiratory belt and fingertip peripheral (SpO_2_) signals from the Skyra scanner, along with, ECG, SpO2, and end‐tidal carbon dioxide (EtCO2) from an anesthesia monitor (Datex‐Ohmeda S/5 Collect software) were measured in synchrony with the MREG/EEG recordings [77].

### 5.3 Preprocessing and Analysis of EEG Data

EEG data were preprocessed using brain Vision Analyzer (Version 2.1; Brain Products) as described in [25]. After correction of the MRI gradient and for ballisto‐cardiographic artifacts, two experienced clinical neurophysiologists (JP, MK) performed consensus sleep scoring according to the 10‐20 system in 30 s epochs, following American Academy of Sleep Medicine (AASM) guidelines for clinical sleep studies (American Academy of Sleep Medicine, 2017). Their viewing of 10–20 min of sleep data from each subject mainly scored as N1 or N2 stage sleep. They also confirmed wakefulness by scoring the awake data, which indicated a single epoch of drowsy N1 sleep in the awake dataset; wakefulness data was missing from two subjects. The present sleep scoring method was consistent with that applied in our previous research [25, 26, 53].

Given our earlier findings that MREG pulsation power increases spatially in the same brain areas as the slow‐delta increases [25], and given the general linear relationship between their powers [40], we wanted to investigate whether the slow‐delta power was also related to the velocity of the pulse in addition to its power. For studying these relationships, we first filtered the EEG data to extract the slow‐delta power (0.2‐2 Hz). We computed the mean of the total slow‐delta power for all subjects, after excluding the noisy EEG channels using procedures implemented in PyPREP Version 0.4.2 [78], mainly consisting of a random sample consensus approach (RANSAC) to identify bad EEG channels [79]. We excluded the EEG data of one subject due to corrupted signal. We then computed the log of the MREG power sum vs. the log of the slow‐delta power sum and filtered out all outliers using the stringent four sigma acceptance threshold, according to Chebyshev's theorem.

### 5.4 Preprocessing of MREG Data

We undertook image reconstruction using a L2‐Tikhonov regularization with lambda value 0.1, where we had determined the latter regularization parameter by the L‐curve method with a MATLAB recon‐tool provided by the sequence developers [80]. Before preprocessing, the image reconstruction also included a critical dynamic off‐resonance in k‐space (DORK) step, which corrected for scanner warming and respiration‐induced dynamic B_0_‐field changes [80, 81].

After reconstruction, we preprocessed and analyzed the MREG data using the Functional MRI of brain Software Library (FSL; Brain Extraction Tool, version 5.09) [82], and Analysis of Functional NeuroImages (AFNI, version 2) [83] in MATLAB (R2021) and Python 3.7.3. We first extracted the brain from structural 3D MPRAGE volumes using neck cleanup and bias field correction options [82]. We then used the FSL pipeline to preprocess the functional data. The pipeline included high‐pass filtering with a cutoff frequency of 0.008 Hz, spatial smoothing with 5 mm full width at half‐maximum (FWHM) Gaussian kernel, and motion correction (FSL 5.08 MCFLIRT) [84]. We found no significant differences between awake and sleep state in the relative or absolute mean displacement values (mm) (p_all_ > 0.094). Nonetheless, we followed the motion correction by applying the 3dDespike function in AFNI (Analysis of Functional NeuroImages, v242) to remove the highest spikes in the MREG data time series. We then registered the preprocessed data to the MNI152 space at 3‐mm resolution, to allow comparable analysis between subjects.

We obtained 10 min of awake data and 10–20 min of sleep data from all subjects. For the final analysis, we used FSL region of interest (fslroi) to segment the data into the best 5‐min awake and sleep epochs, according to the EEG‐verified sleep scoring. For most of the subjects, we used the first 5 min of awake data to confirm wakefulness. For one subject, we chose the last 5 min, because the first 5 min included some sleep, and for another subject we were obliged to use awake data containing an epoch of EEG confirmed sleep. For sleep analysis, we chose a 5‐min epoch that included the highest amount of EEG‐scored sleep (N1 and N2 sleep), as presented in Results.

### 5.5 Respiratory and Cardiovascular Pulsation Ranges

We verified with MATLAB (R2021b) the individual respiratory and cardiac frequency ranges from physiological EtCO_2_ (or respiratory MR scanner belt signals) and fingertip SpO_2_ signals from the FFT spectra of physiological respiratory and cardiac signals c.f. Table 2. We chose the smallest minimum and highest maximum detected respiratory rate values over the whole group (0.08–0.49 Hz). Cardiovascular frequency was high‐passed from 0.51 to the full 5 Hz range for the whole group, to capture harmonics that represent a realistic cardiovascular pulse shape, as required for precise velocity detection [32]. For the very low frequency band, we used the range (0.01–0.08 Hz). We chose the minimum value based on the typical threshold values reported in the literature, and the maximum value on the lowest respiratory values present in our data, thereby minimizing the overlapping effects of respiration and vasomotion. Supplementary materials in [27] showed that MREG and physiological finger‐tip and respiratory belt results had a nearly perfect correlation in awake subjects, but did not correlate well in sleep [85]. Therefore, we did not use MREG to determine the cut‐off frequencies.

**TABLE 2:** Respiratory and cardiac frequency ranges as determined from physiological EtCO_2_ or respiratory belt, SpO_2_ signals, and MREG. Results are expressed as mean ± standard deviation for the peaking values in (*n* = 22) recordings.

Respiratory EtCO_2_ or belt	Peaking value (Hz)
Awake *n* = 22	0.27 ± 0.06
Sleep *n* = 22	0.27 ± 0.05
Cardiac SpO_2_	
Awake *n* = 22	1.02 ± 0.15
Sleep *n* = 22	0.97 ± 0.14
MREG Respiratory	
Awake *n* = 22 Sleep *n* = 22	0.21 ± 0.07 0.26 ± 0.05
MREG Cardiac	
Awake *n* = 22 Sleep *n* = 22	1.03 ± 0.08 0.99 ± 0.07

### 5.6 Optical Flow Analysis

The Lucas‐Kanade multi‐resolution 3D optical flow method has proved to be an efficacious tool for tracking cardiac pulses within the brain [31]. We can thus separately quantify the following parameters: 3D direction $\hat{v}$ and velocity magnitude v_s_, as well as their combined value, i.e., the total velocity $\underset{V}{\to }$ [32]. Specifically, we can compute $\underset{V}{\to }$ as:

(1) $$\underset{V}{\to }=v_{s\_x}\hat{i}\;+v_{s\_y}\hat{j}\;+v_{s\_z}\hat{k}\;=v_{s}\hat{v}$$

where $\hat{v}$ ϵℝ^3^, and **î,** $\hat{j}$, $\hat{k}$ are the three standard unit vectors in the principal x, y, and z directions, respectively.

Every vector in 3D can be represented by the sum of the dot product of its magnitude in the x‐direction multiplied by a unit vector (a vector with magnitude of one cm/s) in the x‐direction, the dot product of its magnitude in the y‐direction multiplied by a unit vector in y‐direction, and the dot product of its magnitude in the z‐direction multiplied by a unit vector in z‐direction. Thus, $\underset{V}{\to }$ can be represented by the dot product of the magnitude v_s_, which equals $|\underset{V}{\to }|=\sqrt{{v_{s\_x}}^{2}+{v_{s\_y}}^{2}+{v_{s\_z}}^{2}}$, and the unit direction $\underline{\hat{v}}$, which equals $\hat{v}=\underset{V}{\to }/\left|\underset{V}{\to }\right|$. In the results section, the $\underset{V}{\to }$ map illustrates velocity as the vector length and its direction, while the v_s_ map illustrates only the combined velocity in 3D.

Each cardiac brain pulse induces a clear MREG signal dip (from momentarily desynchronized spins) that most distinctly arises from major cerebral arteries, including the ACA [7, 9, 28, 32]. This moving cardiac brain pressure pulse was trackable with steps of sparse optical flow analysis [32]. While our previous approach was effective in tracking the cardiac signal (cardiac rate about 80 bpm, i.e., 1.3 Hz), it entailed considerable data loss due to the restricted use of 0.9 s cardiac cycles only. However, this type of restriction of slower periods like respiratory and especially VLF waves brings unacceptable loss of information, because of the greater variability of the slower pulsations due to their physiological signal variability. Our previous study overcame this problem by resampling all the velocity profiles of pulse fronts extracted from the slower respiratory data [33].

The three sources of physiological cerebrovascular pulsations were cardiovascular pulses, vasomotor waves from all cerebral arteries, and respiratory pulses from opposed phase venous blood vs. CSF space pulsations inside the cranium [86]. As liquids do not compress, the pulses immediately transmit the pressure oscillation to perivascular CSF, which then experience phase shifts at a different velocity, depending on the frequency and magnitude of the physiological pulses. While sparse optical flow follows a single moving feature in the data, like the cardiovascular pulse nadir, it has limited capacity to capture displacement features. Here, we resort to dense optical flow analysis, which can track all signal changes between image frames as movement in the human MREG data, and thus captures much finer features of the 3D water movement (c.f. nanonets.com) [35]. In the time domain, dense optical analysis follows all changes over the entire wave pattern, rather than the separately detected signal minima observed in the sparse analysis. In the present study, by using pulsatile water‐flow phantom measurements, we first showed that dense optical flow analysis of MREG data was accurate in flow velocity measurements and then presented dense optical flow results obtained with resampling of all three physiological human brain pulsations.

Given this background, to obtain a more comprehensive summation of the 3D movement of brain pulsations, we used the dense optical flow analysis of MREG data for the cardiovascular, respiratory, and vasomotor frequencies. Then, we resampled the optical flow results of cardiac pulses to 0.9 s and the respiratory pulsations to 6 s, based on our previous studies [32, 33]. For vasomotor waves, we used a 20 s pulse cycle length, based on a pioneering study of vasomotor BOLD wave [87].

For pulse triggering of the three physiological pulsations, we used the intracranial regions of interest with the most conspicuous and powerful pulsations, i.e., the ACA for cardiovascular [32], the 4th ventricle for respiratory [33], and the PCC for vasomotor pulses [87]. Appropriate multi‐resolution pyramid depth was used to compute the velocity, with scaling factors of 3 for cardiac, 1 for respiratory, and zero for the VLF pulsations.

### 5.7 Statistical Analysis of Velocity

We calculated a mean optical flow velocity magnitude (v_s_) map for each subject in 3D MNI space over the 0.9, 6, and 20 s cycles, respectively. We then performed a paired statistical analysis of the v_s_ maps between awake and sleep states using FSL‐TFCE (Threshold‐Free Cluster Enhancement) randomise (threshold *p* < 0.05, *n* = 5000) test sequentially for every time frame of the cycles, i.e., 9, 60, and 200 3D volumes. This yielded statistical maps for, respectively, v_s_card_, v_s_resp_, and v_s_vaso_ over the cycles to provide a dynamic view of the pulsation velocity changes, as in our previous work [33].

We likewise analyzed the directional velocity $\hat{v}$ maps analysis to find the reverse in flow directions in the contrast of sleep and awake states of the subjects. Here, we separated the x‐, y‐, and z‐components of the vectors, and compared separately the positive and negative directions of every component. We combined the resultant maps to depict the reversed $\hat{v}$ between the two states of arousal states as in [33]. We depicted the results in 4 segments within the cycle, overlaid upon 1 mm MNI152 space.

### 5.8 Power Analysis

We utilized the 3dPeriodogram function in AFNI to compute an FFT power density map for each MREG dataset, separately for individuals, and by condition. The total number of time points across all scanning segments was 2861, and we conducted the FFT analysis with 4096 bins, resulting in 2048 bins covering the 0‐5 Hz frequency range. Next, we distinguished the very low frequency (0.01–0.08 Hz) and individual respiratory, and cardiac FFT power frequency ranges from the global and voxel‐wise MREG periodograms. We achieved this by applying fslroi after specifying the relevant individual frequencies, and then calculated the summed power over each frequency range using the 3dTstat function as described previously [25]. The cardiorespiratory pulsation frequencies were also verified using the MR scanner respiratory belt (or anesthesia ETCO_2_ monitor Datex Ohmeda Aesthiva 5) and right index fingertip SpO_2_. For summary results, see Table 2. Finally, we performed a paired statistical analysis of the mean power maps between awake and sleep states using FSL‐TFCE randomise (threshold *p* < 0.05). We applied brain structural segmentation using FSLeyes edit mode and used FSL cross‐correlation (fslcc) to compute the correlation coefficients between power and velocity analysis results.

### 5.9 Flow Velocity Verification Process

We confirmed flow velocity using a phantom consisting of a fresh pineapple (having a striated tissue structure), which had been prepared by drilling a 7 mm diameter hole through the core, through which we pumped water with a peristaltic pump (Watson Marlow 313S) via an elastic tube arising from outside the Faraday cage. The frequency of the pump was not 1:1 related to the number of cycles per second. The peristaltic pump has a rotor with three heads, each pushing the liquid in the same direction during one single cycle. The phantom imaging began with a 1 min baseline recording, followed by recordings at three flow velocity stages (10%, 20%, and 30% of max rotation speed, i.e., 400 rpm), each lasting 1 min. For an illustration of the phantom measurement and the ultrasound velocity verification protocols, c.f. Figure S3.

After scanning the pineapple, we preprocessed the MREG data by subtracting the mean of baseline signal from all other flow‐rate stages. Then, we aligned the MREG data with anatomical T1 & T2 images after motion control and other pre‐processing steps, whereupon we performed sparse and dense optical flow analyses matching those performed on subject data. These data were analyzed with a resolution pyramid of scale 3, because the peristaltic pulsation frequencies were faster than cardiovascular pulses.

## Funding

This work was supported by Uniogs/MRC Oulu DP‐grant (HH), Emil Aaltonen Foundation (HH, MJ, LR), Pohjois‐Suomen Terveydenhuollon tukisäätiö (HH, VKo), The EU Joint Programme – Neurodegenerative Disease Research 2022‐120 (VKi), Jane and Aatos Erkko Foundation grants I and 210043 (VKi), Academy of Finland TERVA grants I‐II 314497, 335720 (VKi), Academy of Finland Grant 275342, 338599 (VKi), Valtion tutkimusrahoitus grants from Oulu University hospital (VKi, VKo), Orion Research Foundation sr (HH, MJ), Finnish Brain Foundation sr (Vki), The Finnish Medical Foundation (VKi, MJ), Paulo Foundation (HH), Maire Taponen Foundation sr (LR), Uulo Arhio Foundation (LR).

## Conflicts of Interest

The authors declare no conflicts of interest.

## Supporting information




**Supporting File 1**: advs74043‐sup‐0001‐SuppMat.docx.

**Supporting File 2**: advs74043‐sup‐0002‐VideoS1.mov.

**Supporting File 3**: advs74043‐sup‐0002‐VideoS2.mov.

**Supporting File 4**: advs74043‐sup‐0002‐VideoS3.mov.

## Data Availability

The data that support the findings of this study are available on request from the corresponding author. The data are not publicly available due to privacy or ethical restrictions.
