# Supplementary material for: Sleep Alters the Velocity of Physiological Brain Pulsations in Humans
Source: Adv Sci (Weinh). 2026 Feb 3;13(19):e03745. doi: 10.1002/advs.202503745 (PMC13045454; doi:10.1002/advs.202503745)
Supplement: Supplementary file 1 — Supporting File 1: advs74043‐sup‐0001‐SuppMat.docx. [file ADVS-13-e03745-s003.docx]

Supporting Information

**Sleep alters velocity of physiological brain pulsations**

*Ahmed Elabasy^*^,Heta Helakari, Tommi Väyrynen, Zalán Rajna, Niko Huotari, Lauri Raitamaa, Ville Isokoski, Matti Järvelä, Mika Kaakinen, Johanna Piispala, Mika Kallio, Vesa Korhonen, Tapio Seppänen, Vesa Kiviniemi^*^*


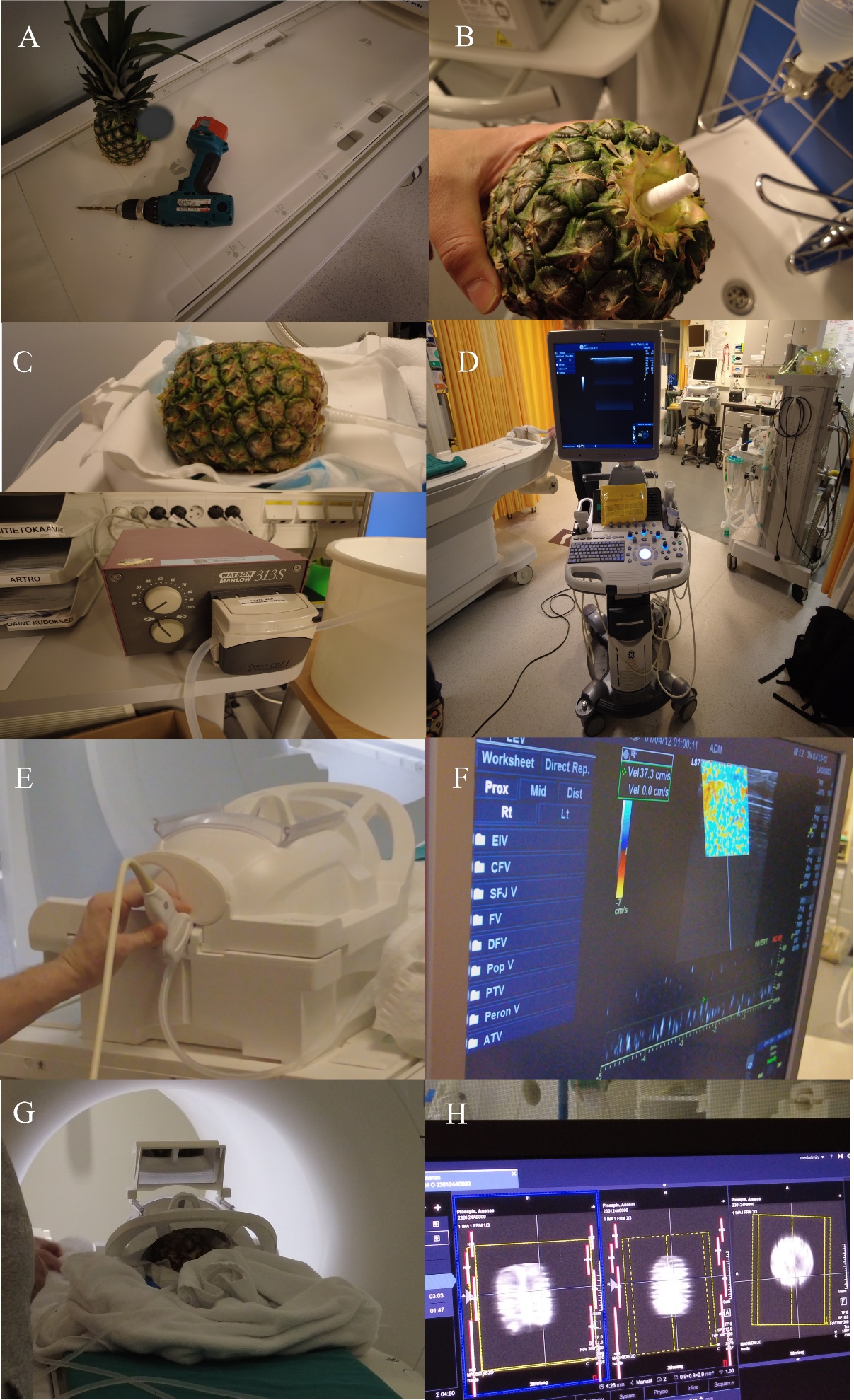


**Supplementary Figure 1.** Scanning a pineapple protocol. (A) We connected the peristaltic pump to the two plastic ends through elastic pipes. (B) Initialization of the ultrasonic meter. (C) Fixation of the meter peripherally to the point of flow input. (D) Initial measurement at (30% maximal pump velocity). (E) Placement of the pineapple in the scanner. (F) Scanning the pineapple using the MREG sequence with the same settings as used for the study subjects.


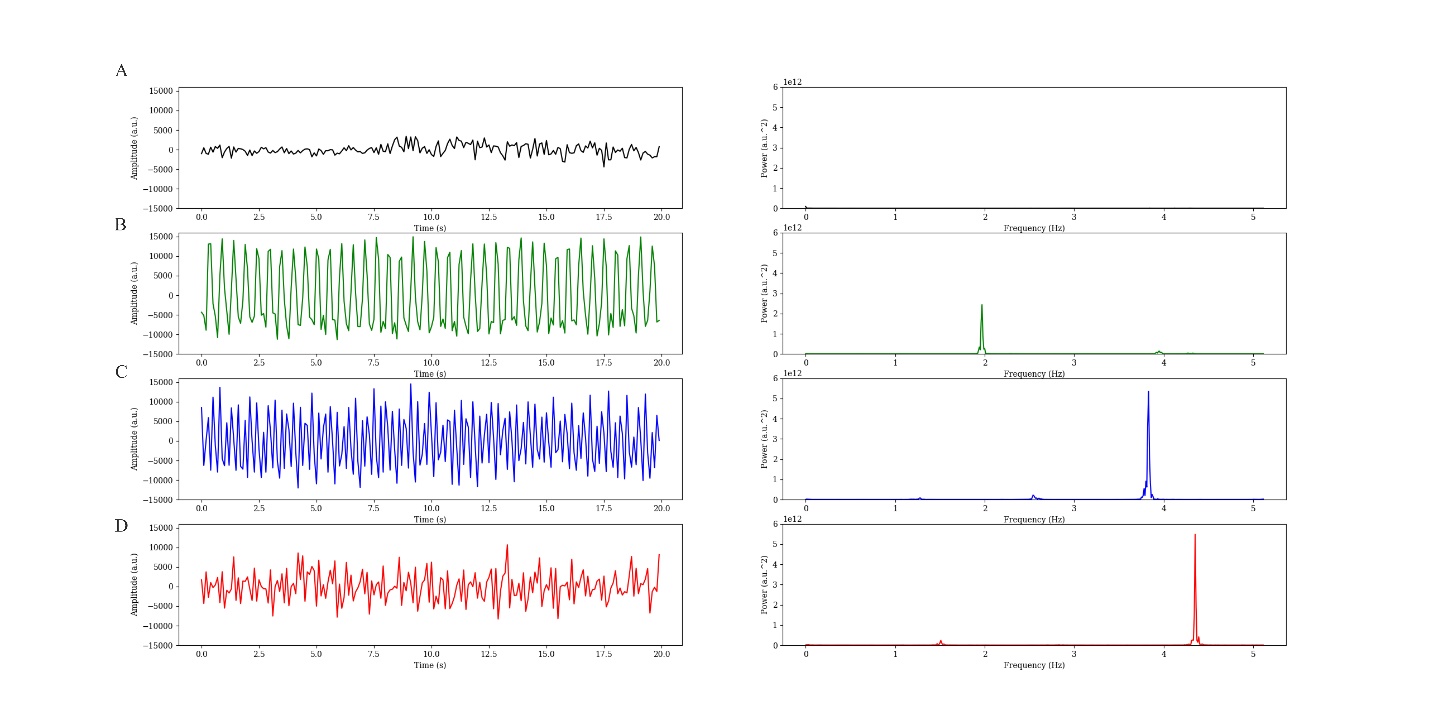


**Supplementary Figure 2.** Time and spectral analysis of the four velocity stages in the pineapple phantom study. (A) Baseline signal. (B) 10% (2 Hz pumping frequency) velocity of the maximal pump velocity. (C) 20% (4 Hz) of the maximal pump velocity. (D) 30% of the maximal pump velocity 6 Hz aliased to 4.3 Hz.


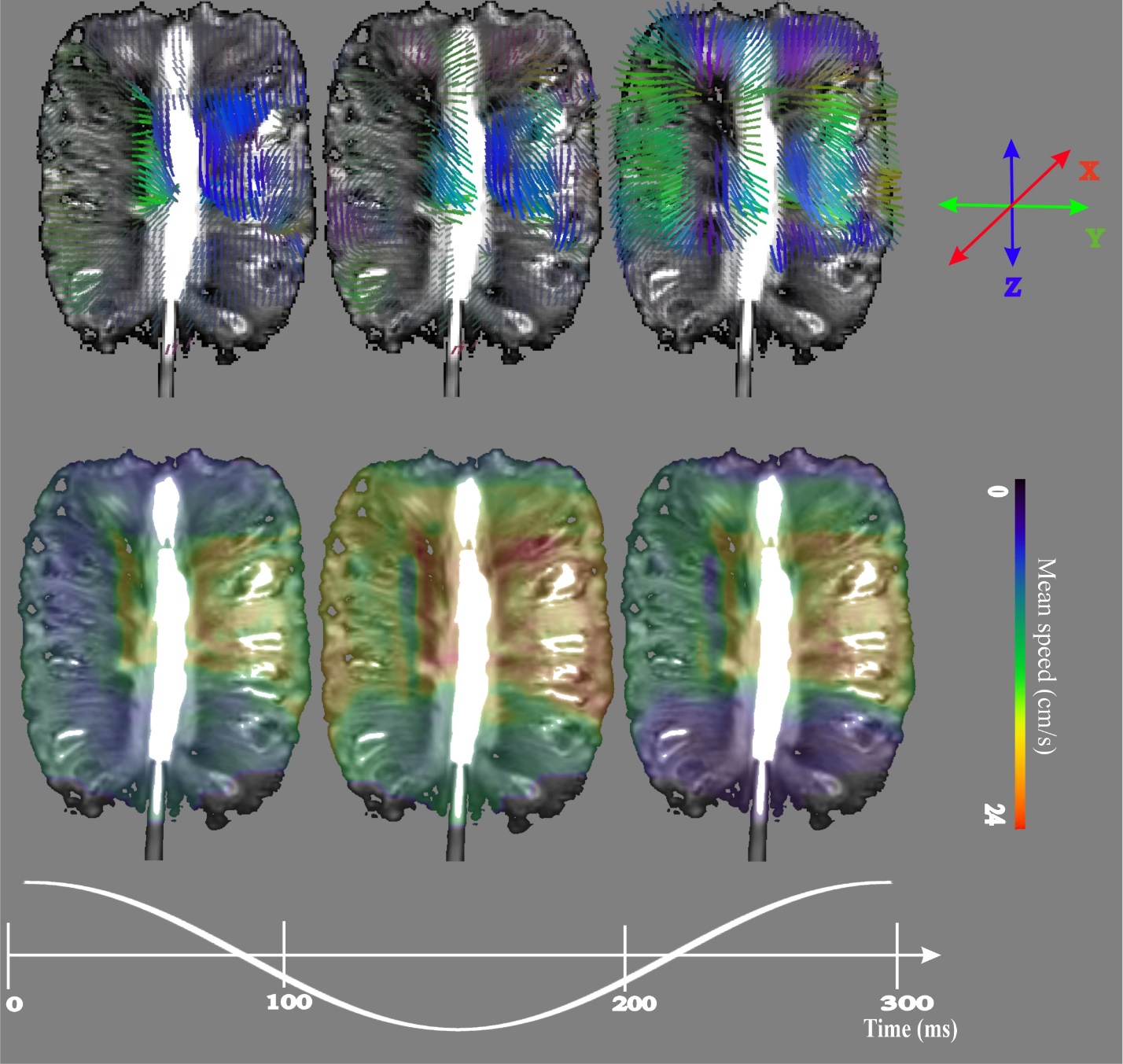


C

B

A

**Supplementary Figure 3.** Mean velocity of dense optical flow analysis at stage 2 (20% maximal flow pump speed) through the pineapple. (A) The 3D directional $\overset{\to}{V}$ maps of the stage 2 water flow pulses are analyzed in three-time segments over the averaged 0.3 s cycle. (B) Velocity magnitude v**_s_** analysis of stage 2, over the three-time segments of the cycle. (C) Representative time domain signal of the stage 2 signal.


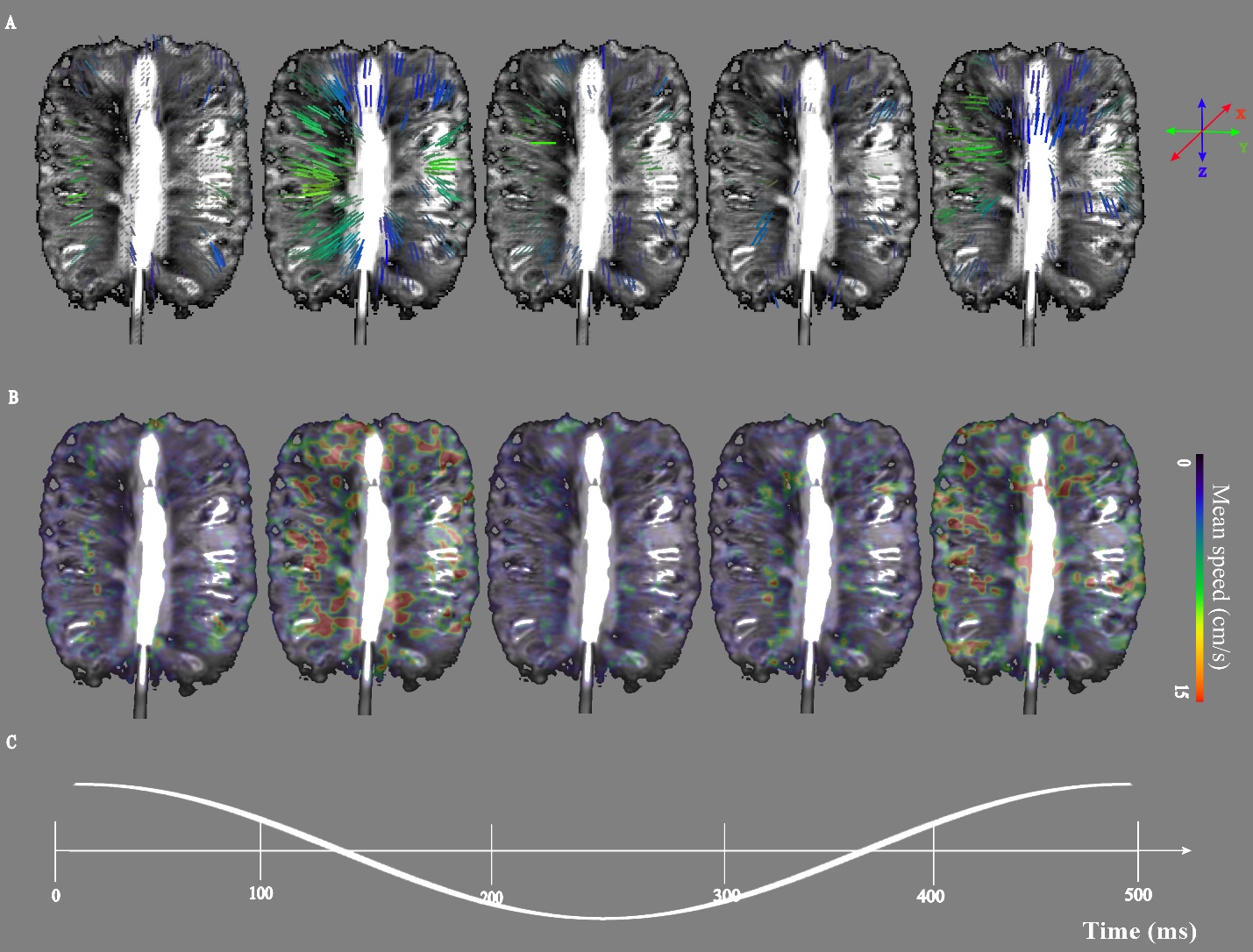


**Supplementary Figure 4.** Mean velocity of *sparse* optical flow analysis at stage 1 (10% maximal flow pump speed) through the pineapple. (A) The 3D directional $\overset{\to}{V}$ maps of the stage 1 water flow pulses are analyzed in five-time segments over the averaged 0.5 s cycle. (B) Velocity magnitude v_s_ analysis of stage 1, over the five-time segments of the cycle. (C) Representative time domain signal of stage 1 signal.

**
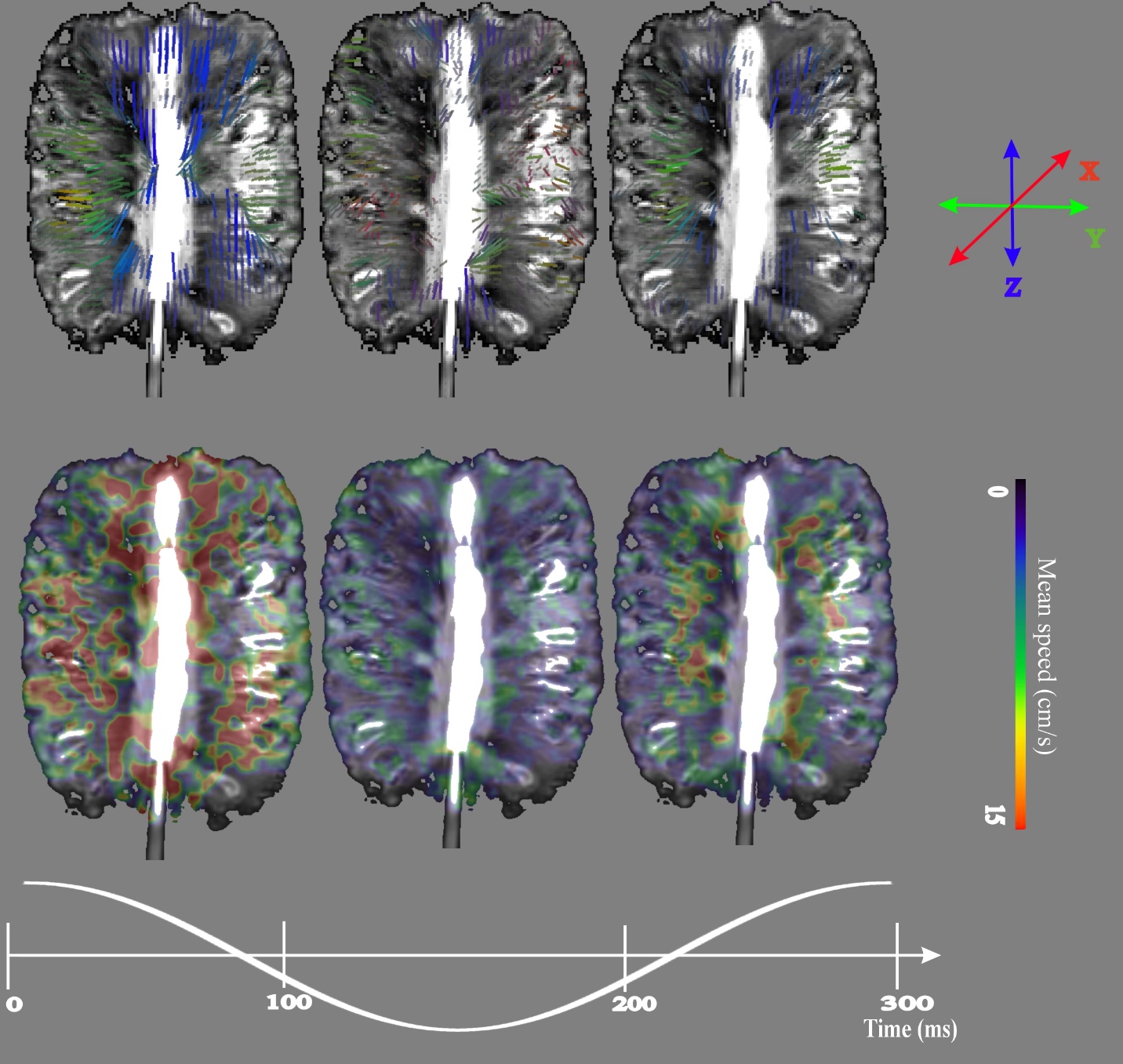
**

C

B

A

**Supplementary Figure 5.** Mean velocity of *sparse* optical flow analysis on stage 2 (20% maximal flow pump speed) through the pineapple. (A) The 3D directional $\overset{\to}{V}$ maps of the stage 2 water flow pulses are analyzed in three-time segments over the averaged 0.3 s cycle. (B) Velocity magnitude v_s_ analysis of stage 2, over the three-time segments of the cycle. (C) Representative time domain signal of stage 2 signal.


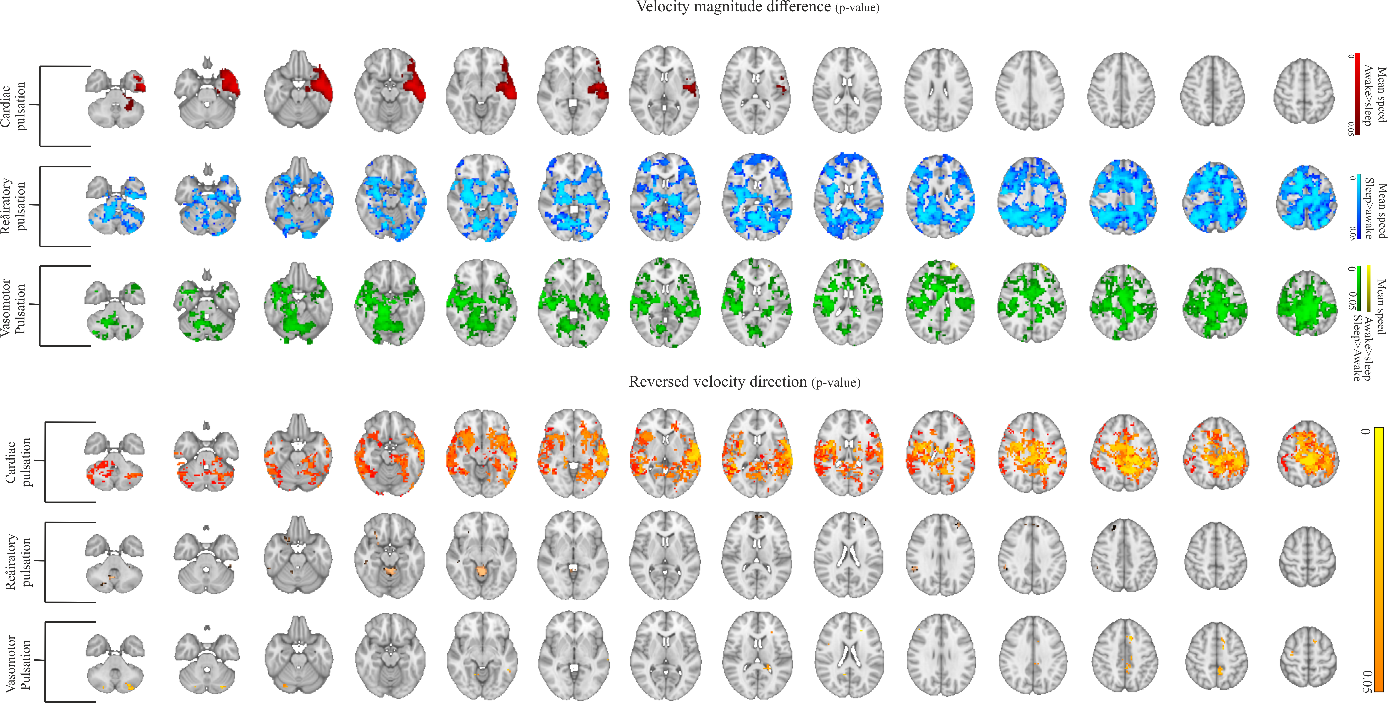


**Supplementary Figure 6**. The upper three rows show the total spatial distribution of significantly altered velocity magnitude in the contrast between sleep (n=22) and awake (n=22) for decreased cardiac, and, increased respiratory and vasomotor pulsations. The bottom three rows show the distribution of significantly reversed velocity directions in the corresponding contrasts between sleep ad awake states. Reversed velocity direction in the comparison between awake (n=22) and sleep (n=22) groups (FSL randomise, family-wise-error-rate (FWER) correction, p<0.05)


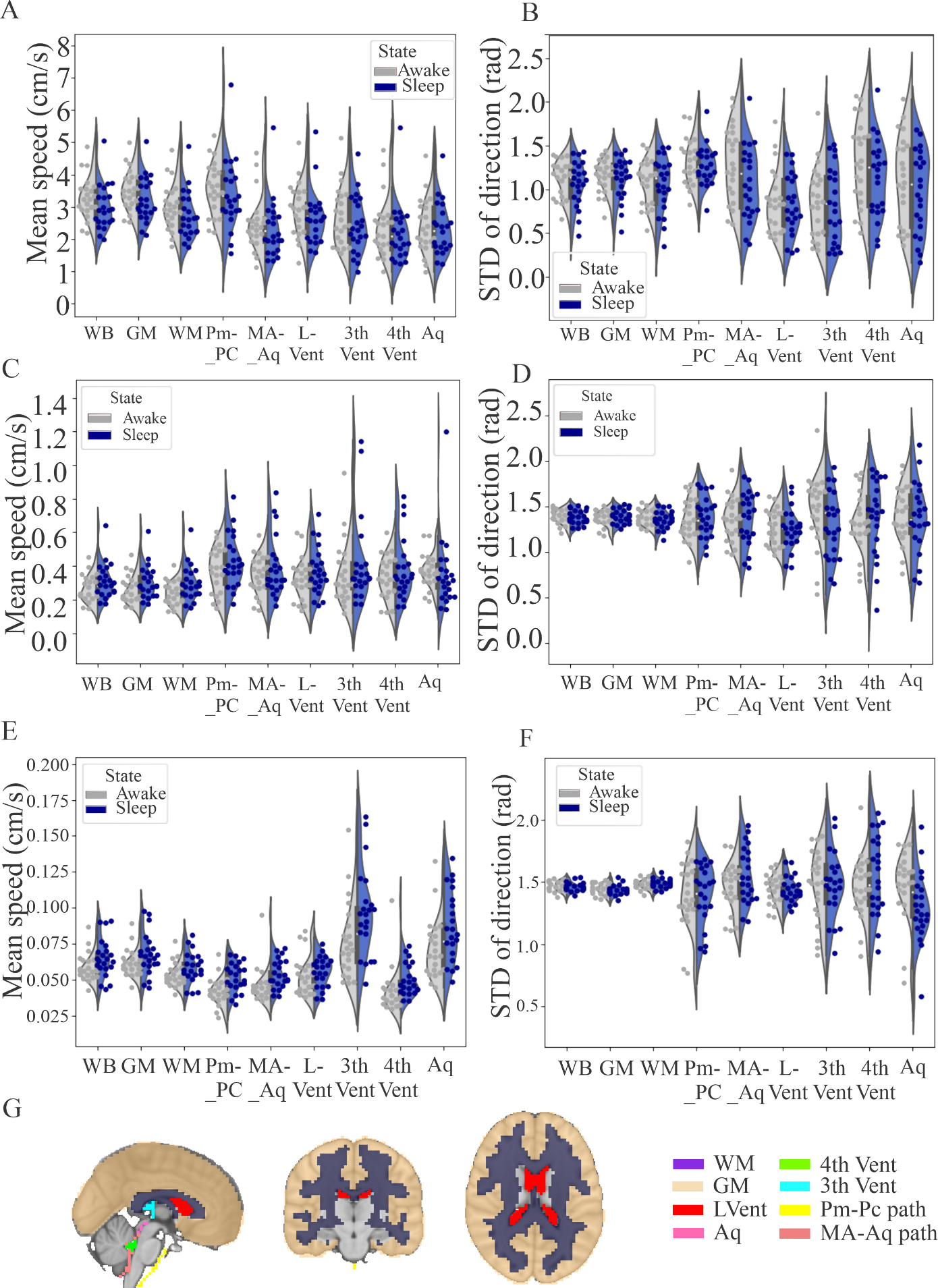


**Supplementary Figure 7**. *(A) Violin frequency plots with group mean cardiovascular v_scard_ (n=22) and (B) the overall directionality STD of* $\mathbf{v̂}$*_card_ in the study subjects (n=22) in awake and sleep states over the whole brain and in segmented brain structures, with individuals indicated as dots. (C) Distribution of mean respiratory v_sresp_ between groups over a complete respiratory cycle in different brain regions, and (D) STD of the direction of* $\mathbf{v̂}$*_resp_. (E) Mean vasomotor v_s_vaso_ velocity of awake and sleeping subjects (n=22 each) extending over a complete vasomotor wave, and (F) STD of* $\mathbf{v̂}$*_vaso_ between groups. (G) The FSL-derived Montreal Neurological Institute (MNI) ROIs are illustrated in three planes (x,y,z); Whole brain (WB), grey matter (GM) and white matter (WM), median aperture (MA), pontomedullary cistern (PM), pontine cistern (PC), all CSF ventricles (Lateral, III, IV) and cerebral aqueduct (Aq) are presented.*


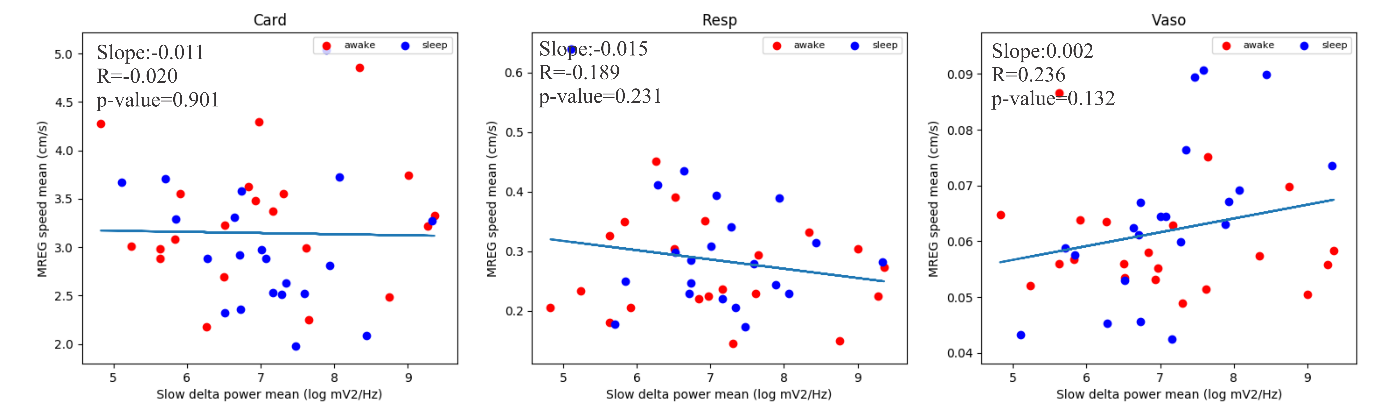

**Supplementary Figure 8.** Velocity and slow-delta EEG power analysis of subjects (n=22) in the two arousal states, i.e. awake and NREM sleep, showing the correlations between brainwide mean propagation velocity of the (from left to right) cardiac, respiratory, and vasomotor wavers to MREG as functions of Delta EEG power in each subject.


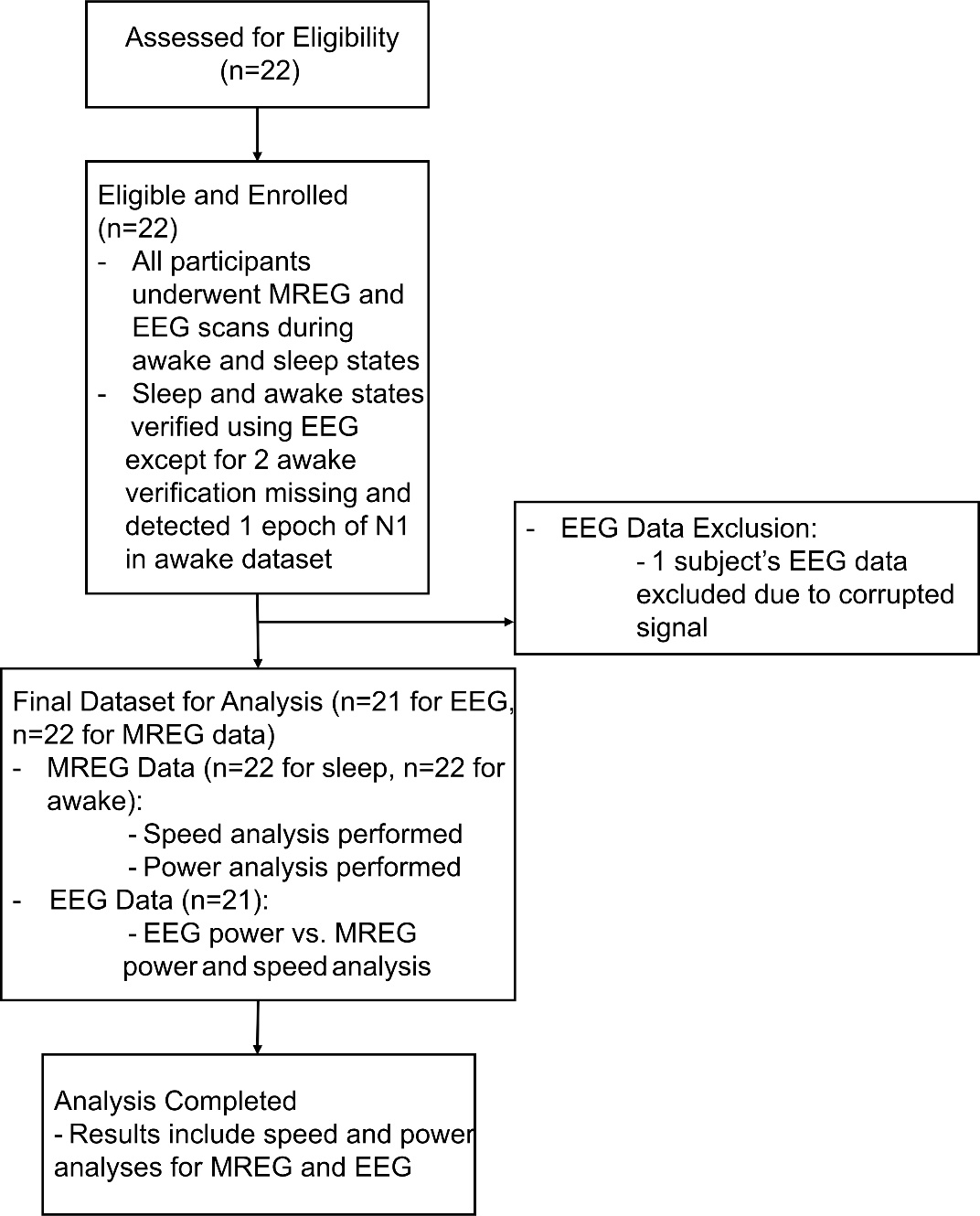


**Supplementary Figure 9.** Study flow diagram.


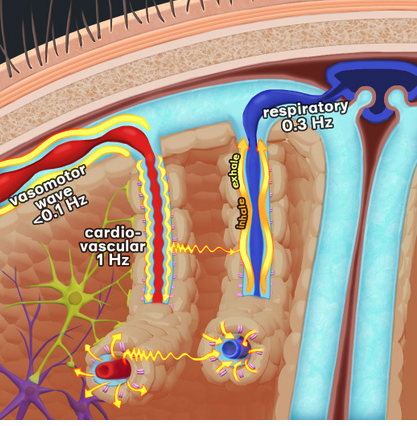


**Supplementary Figure 10.** Physiological pulsations mediate CSF, blood, and intracellular compartments. (copyright for using it is in progress)

[Supplementary video S1](https://drive.google.com/file/d/1wJXUI8foOVUUyWBdkKPKdtfW36lNhy-1/view?usp=sharing): dense optical flow analysis of cardiac BOLD MREG signal [Supplementary video S2](https://drive.google.com/file/d/1vl2l-qS172_jg28DClhJpeIl_TxT0Zo0/view?usp=sharing): optical flow analysis of respiratory BOLD MREG signal [Supplementary video S3](https://drive.google.com/file/d/1rYyICd5XfM--NTdH4EhdtvefGtNk_pPm/view?usp=sharing): optical flow analysis of vasomotor BOLD MREG signal
